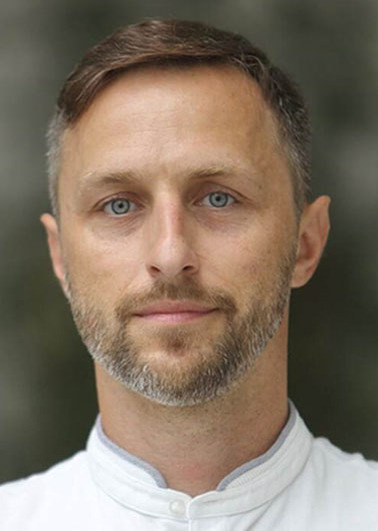# Supported Iridium‐based Oxygen Evolution Reaction Electrocatalysts ‐ Recent Developments

**DOI:** 10.1002/cctc.202200586

**Published:** 2022-08-24

**Authors:** Leonard Moriau, Milutin Smiljanić, Anja Lončar, Nejc Hodnik

**Affiliations:** ^1^ Department of Materials Chemistry National Institute of Chemistry Hajdrihova 19 1001 Ljubljana Slovenia; ^2^ University of Nova Gorica Vipavska 13 5000 Nova Gorica Slovenia

**Keywords:** Iridium, Oxygen evolution reaction• Supported catalysts, Acidic media, Electrocatalysis

## Abstract

The commercialization of acidic proton exchange membrane water electrolyzers (PEMWE) is heavily hindered by the price and scarcity of oxygen evolution reaction (OER) catalyst, i. e. iridium and its oxides. One of the solutions to enhance the utilization of this precious metal is to use a support to distribute well dispersed Ir nanoparticles. In addition, adequately chosen support can also impact the activity and stability of the catalyst. However, not many materials can sustain the oxidative and acidic conditions of OER in PEMWE. Hereby, we critically and extensively review the different materials proposed as possible supports for OER in acidic media and the effect they have on iridium performances.

## Graphical abstract

**Ir‐supported catalysts**: The development of supports able to withstand the OER conditions in acidic PEMWE is of vital importance for the future of hydrogen economy. The various proposed materials to support iridium nanoparticles are summarized here. In addition, their advantages and drawbacks on the catalytic performances are reviewed and analyzed.

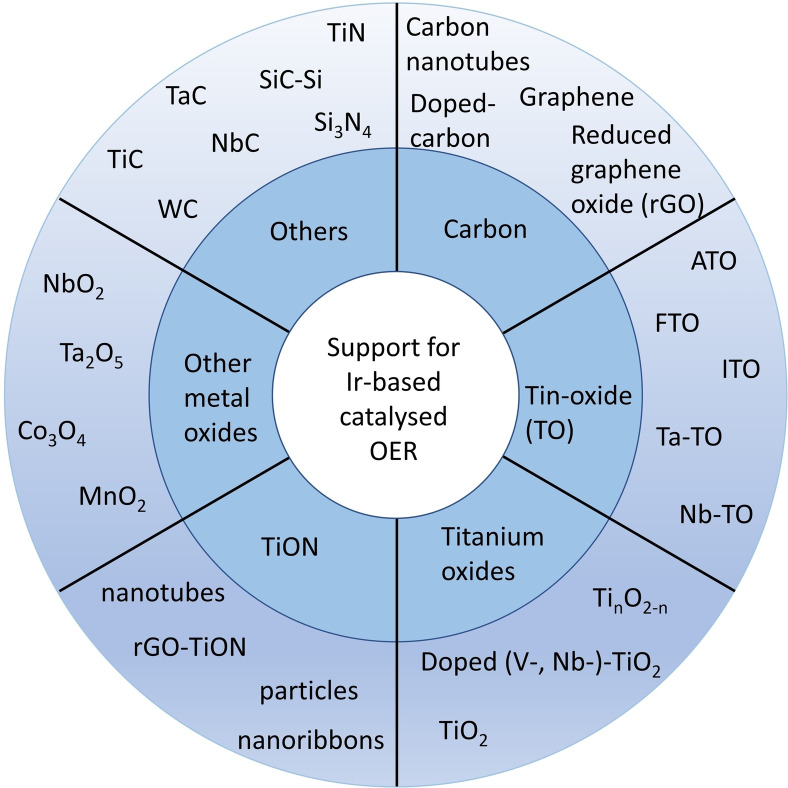

## 1 Introduction

In the COP26 event that took place in November 2021 in Glasgow, the participating parties have again confirmed the Paris agreement to keep global warming under 1.5 °C.^[1]^ This will not be achievable if humanity continues to rely predominantly on fossil fuels as energy sources. Therefore, a greener economy needs to be implemented worldwide. Unfortunately, renewable energy sources such as wind or solar suffer from intermittency and uneven distribution, which is currently making them unreliable. Hence, the sustainable energy transition can only be realized with the aid of efficient energy storage and conversion systems.[^2^, ^3^] Hydrogen is a promising candidate to act as an energy vector, as it enables the storage of electrical energy in the form of chemical bonds. Its key features such as high specific weight energy density (142 MJ/kg), a possible generation with zero carbon emissions, an option of converting it to liquid fuel for easy storage, and safe transportation make hydrogen a versatile and useful energy carrier in the green economy plan.[^4^, ^5^] Presently, almost all of the H_2_ is produced via energy demanding and CO_2_‐emitting methods such as methane reforming, coal gasification, or biomass conversion.[^6^, ^7^] In order to meet the carbon‐free criteria, its production should be directed towards cleaner processes such as electrochemical (or photocatalytic) splitting of water into O_2_ and H_2_ in a water electrolyzer (WE) device.[^8^, ^9^, ^10^, ^11^]

Commercial WEs already exist and are classified based on the pH of the electrolyte used, namely alkaline and acidic water electrolyzers.[^12^, ^13^] While alkaline electrolyzers, which use more abundant and less expensive materials, notably for catalysts are already commercially available on a large scale,[^14^, ^15^] acidic proton‐exchange membrane electrolyzers (PEMWE) present the advantages of higher ionic conductivity and lower ohmic losses, shorter start‐up times, higher efficiency (higher power density and current density), less side reactions and lower crossover of gases.[^12^, ^16^, ^17^, ^18^] Therefore, PEMWEs have attracted growing attention for the coupling with intermittent sun and wind energy sources and are being widely considered as a cornerstone for the future hydrogen economy.^[11]^

In both kinds of WE (alkaline and acidic), the hydrogen evolution reaction (HER) takes place at the cathode while the oxygen evolution reaction (OER) occurs at the anode. OER is a four‐electron transfer reaction while HER involves the transfer of only two electrons,[^11^, ^19^, ^20^, ^21^] which makes it considerably faster than OER. Therefore, OER is the limiting reaction for the efficiency of PEMWE. To accelerate the kinetics of reactions, catalysts are used on both electrodes. In the case of HER, a low amount of platinum deposited on high surface area carbon support is sufficient to reach excellent performances.[^22^, ^23^, ^24^] For OER, the state‐of‐the‐art catalysts are precious metals belonging to the platinum group metals (PGMs) and gold. Among the PGMs, the activity for OER in acidic media is following the trend Ru>Ir>Rh>Pd>Pt>Au.[^20^, ^25^, ^26^, ^27^] On the other hand, the stability follows a different trend with Pt>Rh>Ir>Au>Ru.[^25^, ^28^] Ru is thus the most active catalyst for OER, but also the least stable. It suffers from high dissolution due to the formation of soluble RuO_4_^−^ during OER.[^25^, ^29^] Therefore, Ir and its oxides are widely considered the state‐of‐the‐art catalysts for OER, in both acidic and alkaline media, as they offer the best balance between activity and stability.[^11^, ^20^, ^30^, ^31^, ^32^, ^33^]

Unfortunately, iridium is one of the rarest elements with a presence of 0.001 ppm in the Earth ‘s crust^[34]^ and with less than 10 T mined per year.^[35]^ Currently, water electrolyzer would require 250–500 kg_Ir_/GW for a power density of 4 W/cm^2^ with a loading of 1–2 mg_Ir_/cm^2^.^[36]^ Some predictions, based on different Ir loadings, planned that 0.4 to 5.36 T_Ir_/year will be needed to increase the grid capability by 8 GW/year,^[37]^ while the global energy demand is projected to increase by 24–26 TW in 2040,^[38]^ with around 12 % accounting for the electricity sector.^[41]^ Another study predicts that in 2030, a quarter of the current worldwide Ir production would be required just to meet the PEMWEs demand in Germany.^[42]^ In any case, a drastic reduction of iridium utilization in WE is needed to allow for a widespread implementation of the technology, a 10‐fold decrease would be a minimum according to Babic et al.^[36]^ However, this decrease should not impact the performance of WE. Ideally, improved performances should be obtained while decreasing the Ir loading.[^28^, ^41^] Thereby, a power density of 10 W/cm^2^ (2.5 times improvement) with a loading of 0.5 mg_Ir_/cm^2^ (4‐fold decreased) would require only 50 kg_Ir_/GW and could be even reduced to 10 kg_Ir_/GW with longer‐term development of the WE technology.^[36]^

To reach these objectives, several strategies are envisaged. They can be separated into two different approaches: increasing the number of active sites present in the catalyst and improving the activity of each active site.[^38^, ^42^, ^43^, ^44^, ^45^] Both have already been proven for various electrocatalysts and reactions.[^46^, ^47^, ^48^, ^49^, ^50^]

**Figure 1:**
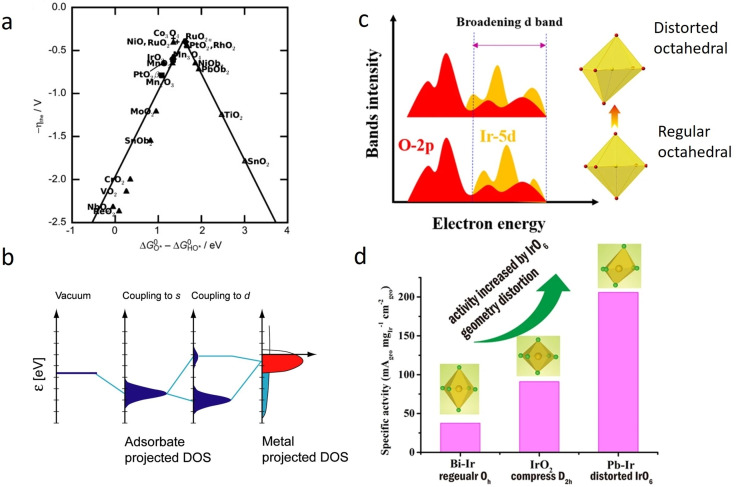
a) Activity trend towards oxygen evolution for various oxides. Reproduced with permission.^[53]^ Copyright 2011, Wiley‐VCH. b) Schematic illustration of bond formation at a transition‐metal surface. Reproduced with permission.^[55]^ Copyright 2011, National Academy of Sciences. c) Schematic diagram of the broadening Ir‐5d band induced by distorted octahedra, enhancing orbitals overlap between Ir d‐ and O‐2p bands.^[58]^ Open access, 2016. d) OER specific activity order of IrO_2_, Bi−Ir and Pb−Ir in accordance with the order of IrO_6_ geometry distortion.^[58]^ Open access, 2016.

Improving the activity of each active site comes by optimizing the interaction between the active compound and the reactants and products.^[38]^ The activity of material towards an electrochemical reaction is linked to its ability to bond with the reactants and its capability to release the products. For both, the binding should not be too strong nor too weak as established by Sabatier.^[51]^ An optimal catalyst should moderately bind both intermediates. Based on this, a volcano plot can be constructed. In the early 80s, Trasatti showed that the empirically measured activity for OER followed a volcano‐plot relationship constructed on the standard enthalpy of the transition from lower to higher oxide‐form.^[52]^ Nowadays, the difference in the free binding energy between O* and OH* is the commonly used catalytic descriptor for OER volcano‐plot (Figure 1a).^[53]^ In addition to the different materials, it was also shown that the type of oxide, e. g. rutile, spinel, or perovskite, influences the activity.[^20^, ^53^] For example, rutile type RuO_2_ is more active than its spinel counterpart.[^20^, ^53^] The d‐band position of a material affects the adsorption strength. For example, if the d‐band is shifted towards a lower level, the occupation of the anti‐bonding orbital formed during the reaction is increased and thus the adsorption of OER intermediates on the metal active sites is weakened^[54]^ (d‐band theory shown in Figure 1b^[55]^). Similarly, inducing a lattice strain near the active site, e. g. by creating a vacancy, will modify the electronic structure and thus the adsorption strength of the adsorbate.[^56^, ^57^] The lattice strain effect was utilized to increase the activity of Ir‐based pyrochlore (A_2_B_2_O_7_ materials) towards OER as seen in Figures 1c and d.^[58]^ Therefore, the activity of an individual active site can be tuned with a careful design/modification of its electronic environment.

Introducing foreign atoms by alloying or doping the noble metal (Ir or Ru) with less noble metal (i. e., transition metal as Ni, Co, …) shifts the d‐band position and consequently the energy of the bond to the OER intermediates.[^43^, ^44^, ^59^, ^60^, ^61^, ^62^] It can also induce lattice strain due to the lattice mismatch and thus further tune the electronic structure.[^63^, ^64^] Another parameter influencing the activity is the degree of crystallization of the catalyst. Indeed, it was shown that crystalline RuO_2_ is less active than its amorphous counterpart.[^20^, ^53^] This could be due to different mechanisms for OER on the two materials or a difference in active sites.[^16^, ^65^] The same was found to be true for IrO_2_.[^66^, ^67^, ^68^, ^69^] On the other hand, a higher crystallinity induces higher stability against dissolution.[^70^, ^71^] Therefore, different treatments (thermal vs electrochemical oxidation[^31^, ^72^]) or synthesis were studied to obtain the best activity/stability ratio for the material.[^73^, ^74^]

The second approach is to increase the number of active sites by optimizing the morphology of the catalyst (control of the shape^[75]^ or morphology,^[76]^ porous structure,^[77]^ self‐supported catalyst^[78]^) or by the deposition of nanoparticles of the active material on a high‐surface‐area support.^[79]^

**Figure 2:**
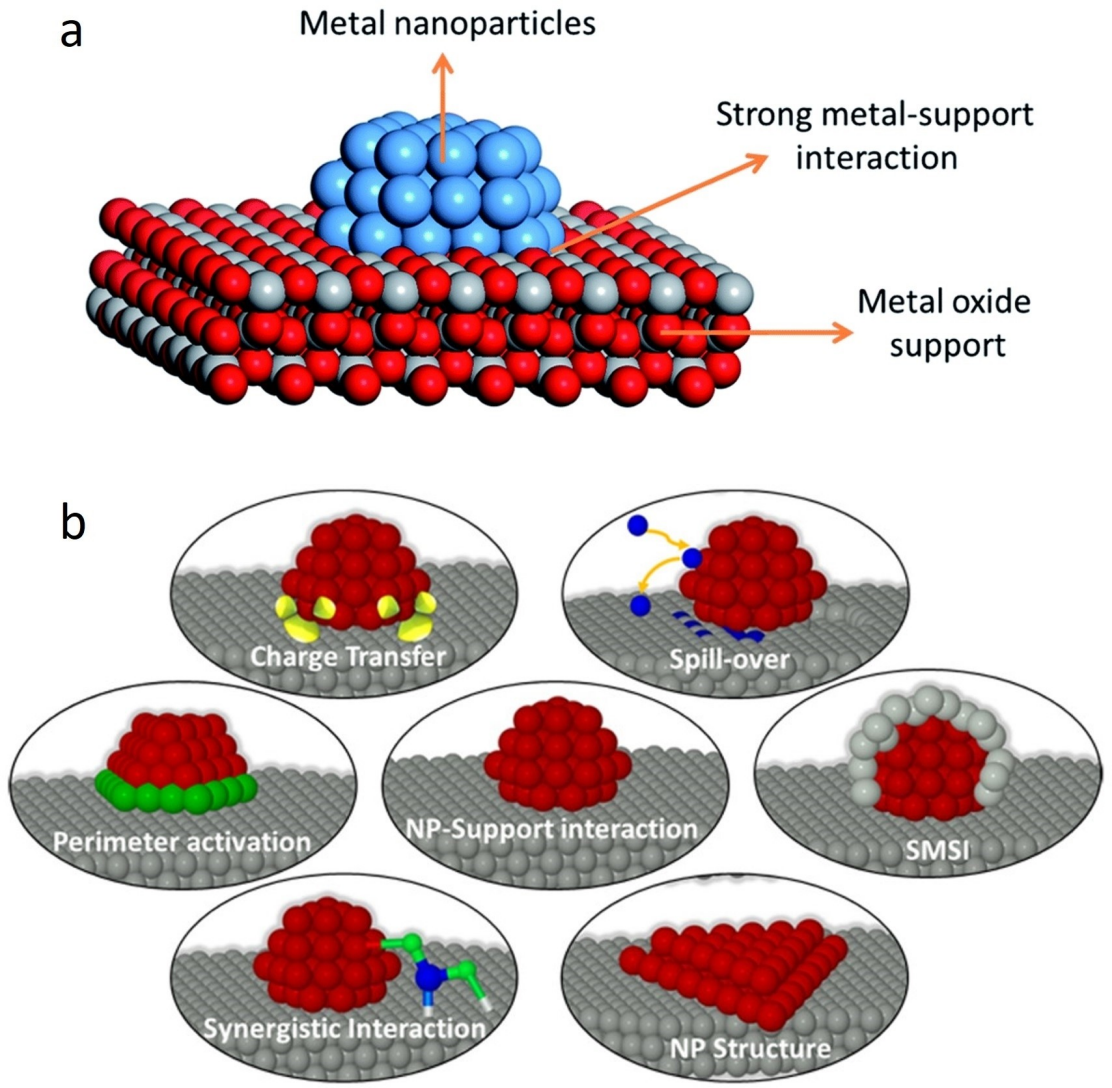
a) Schematic representation of the strong metal‐support interaction (SMSI). Reproduced with permission.^[80]^ Copyright 2020, The Royal Society of Chemistry. b) Possible effects of the support on the deposited nanoparticles. Reproduced with permission.^[81]^ Copyright 2016, American Chemical Society.

Preferably, both strategies should be applied simultaneously, therefore increasing the number of active sites as well as their intrinsic activity.^[38]^ For example, the utilization of support allows the dispersion of catalytic material into smaller nanoparticles, thus increasing the number of active sites compared to the unsupported catalyst for a fixed precious metal mass. On the other hand, it can also affect the chemical environment of the active site. Consequently, the d‐band can be shifted, similarly to alloys or vacancies, and the intrinsic activity can be influenced by the support. Support can also dictate the stability of the catalytic composites through stronger anchoring of metallic nanoparticles. Figure 2 shows the schematic of strong metal‐support interaction (SMSI) between a support and the nanoparticles as well as the possible effects deriving from it.[^80^, ^81^]

Some excellent reviews have covered many aspects of the oxygen evolution reaction, from general ones[^82^, ^83^, ^84^] to more focused on PEMWE,[^18^, ^28^, ^85^, ^86^, ^87^, ^88^] or specialized in catalyst design,[^89^, ^90^, ^91^] mechanism and stability,[^68^, ^92^, ^93^] self‐supported,^[78]^ perovskite,^[94]^ non‐noble,[^95^, ^96^, ^97^, ^98^] etc, while surprisingly none has focused on the proposed supports in an acidic environment. Due to their future importance for the development of WE, we herein extensively review the studied supports for iridium and gather valuable insights into their impact on the catalysts’ activity and stability under OER conditions. First, different materials are discussed based on their material class. Afterwards, the current challenges and directions of research on the development of adequate support are discussed.

## 2 Proposed supports for Ir nanoparticles

Adequate electrochemical support needs to fulfill several requirements, namely it has to exhibit sufficient conductivity, stability under the conditions of the reaction, and ability of deposition of the active compound (iridium in this case). In addition to allowing better utilization of the precious metal in the form of nanoparticles, the support can also influence the performance of the latter.^[99]^ The metal‐support interaction modifies the electronic structure of the active site^[100]^ and thus the activity towards OER. Moreover, it can also suppress the dissolution of nanoparticles or Ostwald ripening and thus improve the stability.^[101]^

### 2.1 Carbon‐based supports

One of the most widely used materials for the dispersion of electrocatalysts is carbon. Its high electrical conductivity and large surface area make it a suitable support for many electrocatalysts, notably for Pt in the case of oxygen reduction reaction in fuel cells.^[102]^ However, it is not expected to be thermodynamically stable under oxygen evolution conditions and thus the stability of the catalyst should be carefully considered when using carbon‐based support.[^93^, ^103^]

**Figure 3:**
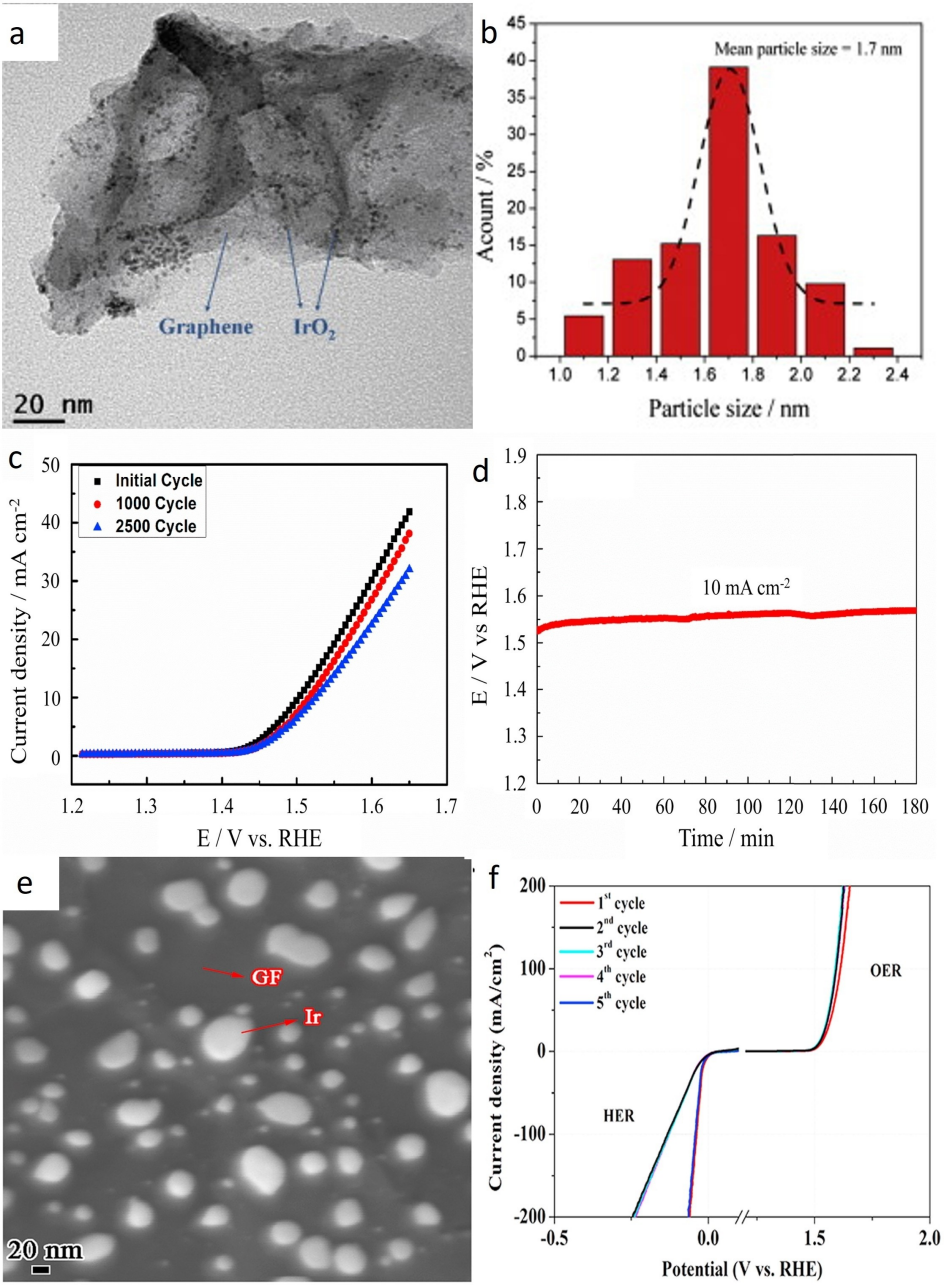
a) TEM picture of IrO_2_/RGO.^[105]^ b) IrO_2_ particle size distribution in IrO_2_/RGO. Reproduced with permission.^[105]^ Copyright 2013, Elsevier. c) Current density changes of linear sweep voltammogram on the IrO_2_/CNT catalyst during durability test.^[106]^ (d) Chronopotentiometry measurement of IrO_2_/CNT at 10 mA/cm^2^. Reproduced with permission.^[106]^ Copyright 2018, Elsevier. e) SEM picture of Ir/GF.^[109]^ f) Alternate HER and OER polarization curves of Ir/GF. Reproduced with permission.^[109]^ Copyright 2017, Elsevier.

One of the first examples, where carbon was used as a support for Ir catalyst in acidic media was presented by Kong et al.[^104^, ^105^] After a study of Pt/Ir on carbon to be used in an unitized regenerative fuel cell (URFC),^[104]^ Kong and his co‐worker focused on pure OER catalysts. Thereby, they synthesized ultrafine IrO_2_ nanoparticles (around 1.7 nm) on a reduced‐graphene oxide (RGO) support as seen in Figures 3a and 3b.^[105]^ The mass activity of the supported catalyst was 2.3 times higher than the activity of commercial benchmark IrO_2_, which was attributed to the enhanced surface area as well as the better electrical conductivity provided by the graphene support. In addition, the IrO_2_/RGO catalyst displayed unexpectedly high stability after 5000 cycles under OER conditions (26.4 % loss vs 38.4 % for the IrO_2_ benchmark). The authors attributed this long‐term stability to the interaction between the π‐electrons of RGO and IrO_2_ which prevents the agglomeration of nanoparticles due to strong anchoring. However, the stability of the support itself, which would be expected to oxidize under these conditions, was not assessed. Similarly, IrO_2_ nanoparticles supported on multi‐walled carbon nanotubes (MWCNT‐IrO_2_) were found to outperform the activity and stability of IrO_2_ benchmark and IrO_2_ on graphene from Kong et al.^[106]^ The 40‐times better activity and longer durability compared to the benchmark were ascribed to the higher surface area of the supported catalyst as well as its interaction with the less electronegative support. This interaction evidenced by the shift of the Ir4 f peak in X‐ray photoelectron spectroscopy (XPS), modified the interaction with OER intermediates and enhanced the electron transport during OER. The high durability of the catalyst (80 % activity retained, Figures 3c and 3d) was ascribed to the mitigation of agglomeration of supported IrO_2_ nanoparticles, oppositely to the unsupported IrO_2_ benchmark.

Later, Ir nanoparticles deposited on carbon‐based support have mostly been investigated as bifunctional catalysts.[^107^, ^108^, ^109^, ^110^, ^111^] Zhang et al. obtained impressive HER and OER catalysts by depositing low content of Ir (5.91 wt%) on 3D‐graphite foam support (Ir/GF) (Figure 3e).^[109]^ The interaction of iridium with the graphite foam support improved not only the OER activity but also the HER activity (Figure 3f), surpassing the Pt/C, the benchmark for the latter reaction. A cell voltage of 1.55 V_RHE_ was obtained for a 10 mA/cm^2^ current in a two‐electrode electrolyzer. In addition, the catalysts could maintain a quasi‐similar activity after 10 hours of electrolysis at 10 mA/cm^2^. However, this experiment was not performed in a real device where the harsher conditions, notably higher temperature, could be more damaging to the carbon. Iridium nanoparticles deposited on vertically grown graphene (Ir/VG) by Roy et al. also show increased performances for OER and HER.^[111]^ The HER activity was similar to Pt/C in an acidic environment while the OER‐benchmark IrO_2_ was outperformed by 30 mV (overpotential of 300 mV vs 330 mV to reach a constant current of 10 mA/cm^2^). The stability of the catalyst was investigated and compared to Ir deposited on plane graphene (Ir/GC). The Ir/VG sample maintained the same activity after 1000 cycles while Ir/GC showed significant degradation. The enhanced stability was assigned to the strong interaction between VG and Ir which prevents the dissolution of metallic iridium. Once again, the stability closer to real‐device conditions, where carbon corrosion is enhanced, was not performed.

**Figure 4:**
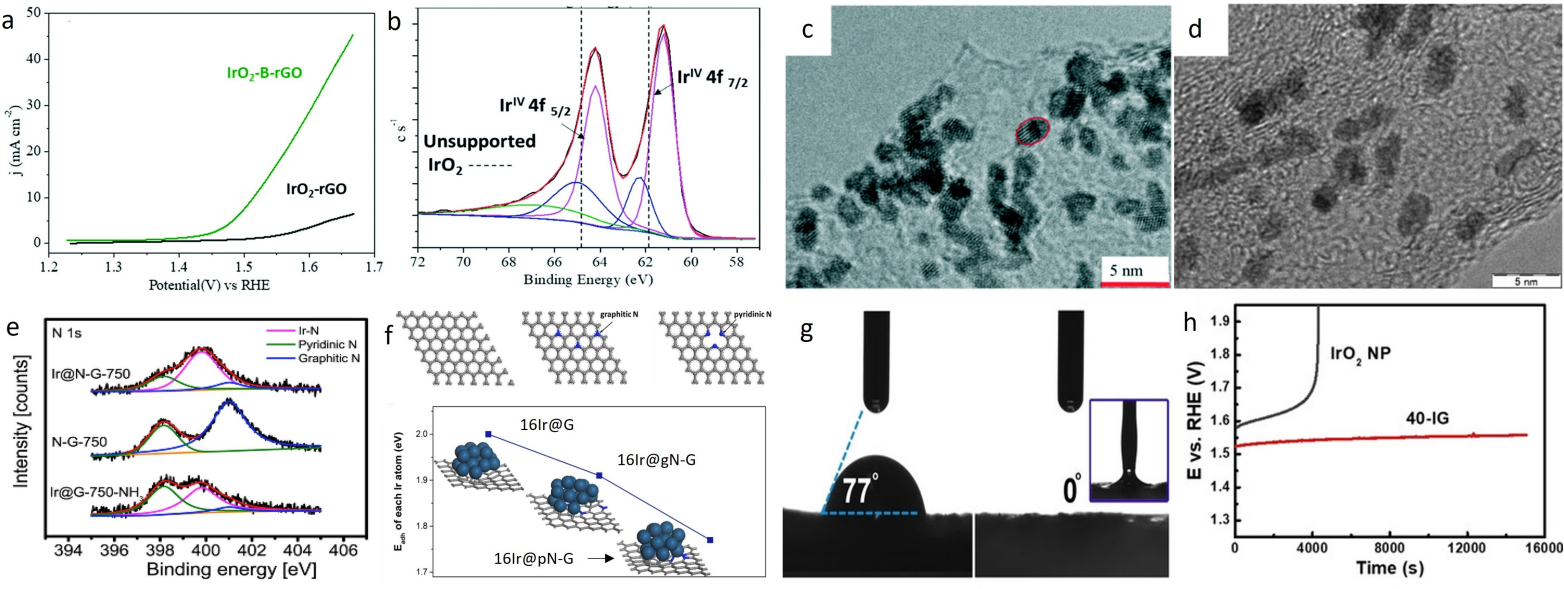
a) Linear sweep voltammetry of IrO_2_−rGO and IrO_2_−B−rGO in 0.5 M H_2_SO_4_ between 1.2 and 1.65 V_RHE_.^[118]^ b) Ir4 f XPS spectra for IrO_2_−B−rGO.^[118]^ c) High resolution (HR)−TEM of IrO_2_−B−rGO before degradation test.^[118]^ d) HR‐TEM of IrO_2_−B−rGO after degradation test. Reproduced with permission.^[118]^ Copyright 2020, The Royal Society of Chemistry. e) High‐resolution N1s XPS spectra of Ir@N−G‐750, N−G‐750, and Ir@G‐750 after the NH_3_ process.^[117]^ f) Up, top view model of, left to right, pristine graphene, graphitic N‐doped graphene and pyridinic N‐doped graphene. Bottom, variation of calculated adhesion energy of Ir on the different substrates. Reproduced with permission.^[117]^ Copyright 2019, Elsevier. g) Contact angle measurements of IrO_2_ nanoparticles (left) and IrO_2_/GCN (right).^[120]^ h) Chronopotentiometry of IrO_2_ and IrO_2_/GCN at 20 mA/cm^2^. Reproduced with permission.^[120]^ Copyright 2019, Wiley‐VCH.

Graphitic carbon appears to present a better resistance towards oxidation than more amorphous forms.[^112^, ^113^, ^114^] In addition, morphological or chemical modification of the graphitic carbon could further improve its stability.^[115]^ Therefore, several modified‐carbon supports have been used to deposit Ir nanoparticles.[^106^, ^110^, ^111^, ^116^, ^117^, ^118^, ^119^, ^120^] For example, Joshi et al. presented iridium nanoparticles sitting on boron‐doped reduced‐graphene support (IrO_2_−B−rGO) with enhanced stability and activity.^[118]^ Their catalyst with around 2.3 wt% boron was compared with Ir nanoparticles (19.6 wt%) of the same size deposited on undoped‐reduced graphene oxide (IrO_2_−rGO). IrO_2_−B−rGO presented an 8‐times higher current density at 1.65 V_RHE_ vs the undoped catalyst (Figure 4a), a better Tafel slope (124.8 mV/dec vs 176.5 mV/dec) as well as a lower onset potential (1.44 V_RHE_ vs 1.54 V_RHE_). The improved activity was attributed to the presence of the boron in the support. The doping has two effects on the graphene support: the opening of the bandgap of the graphene lattice and a strong metal‐substrate interaction between IrO_2_ and B−rGO sheets as proven by theoretical calculations^[121]^ and XPS experiments (Figure 4b). These modified interactions between Ir nanoparticles and the support led to improved activity. In addition, better stability was observed for IrO_2_−B−rGO after cycling between 1.2 and 1.6 V_RHE_. Indeed, even after 1200 cycles, the activity had only slightly decreased while 1000 cycles were enough to lose all OER activity for IrO_2_−rGO. The authors observed a similar trend during the chronoamperometry degradation test. They attributed the higher durability of the catalyst to the synergy between the IrO_2_ nanoparticles and the boron‐doped reduced graphene support, which prevents the loss of the precious metal particles′ surface area as supported by transmission electron microscopy (TEM) images after accelerated degradation test (ADT) (Figures 4c and 4d). This synergy also helps to mitigate the oxidation of the support to CO_2_. The explanation for the carbon stability under harsh oxidizing conditions is that in absence of water, the carbon corrosion cannot happen.[^122^, ^123^] Therefore, if all the water is split faster than the oxidation of carbon, the support can sustain highly oxidizing conditions. This concept is already in use to increase the stability of carbon in proton exchange membrane fuel cells (PEMFC). Therefore, highly active IrO_2_ sites on carbon‐based support would promote water oxidation over carbon corrosion reaction and thus increase support durability. In absence of doping (IrO_2_−rGO) and with lower activity, the protection of the support was not occurring, and big performance losses were observed.

On the other hand, Wu et al. have synthesized small Ir nanoparticles (1.8–2.2 nm) on a nitrogen‐doped graphene support.^[117]^ The catalyst with the support annealed at 750 °C, namely Ir@N−G‐750, presented the best electrochemical performances and was compared with an undoped catalyst, Ir@G‐750. Alike the work of Joshi et al. and their boron‐doped support,^[118]^ the electrocatalyst with doped support presented better performances than its undoped counterpart. Specifically, an overpotential of 303 mV was necessary to achieve a constant current of 10 mA/cm^2^ during OER while 318 mV and 369 mV were needed for Ir/C and Ir@G‐750, respectively. In terms of stability, the same trend as for other doped/undoped catalysts appeared. In this case, Ir@N−G‐750 sustained 20 h at 20 mA/cm^2^ or 2000 cycles while in Ir@G‐750 and Ir/C benchmark significant deterioration occurred. The morphological and chemical stability of the catalyst was confirmed by XPS and TEM. XPS pointed out the interaction between iridium particles and the nitrogen present in the support, mostly on pyridinic sites (Figures 4e and 4 f). To understand the reason for these improved performances, the authors used density functional theory (DFT) in addition to characterization methods (XRD, XPS and TEM). The interactions between the two elements in the pyridinic sites of the support helped boosting iridium stability as well as activity. The DFT calculations further confirmed that Ir_16_ clusters were stabilized by coordinating N‐dopant.

At the same time, Chen et al obtained one of the best mass‐activity recorded for an Ir‐based catalyst in acidic media.^[120]^ Their IrO_2_ nanoparticles on graphitic carbon nitride (IrO_2_/GCN) catalyst reached an impressive mass‐activity of 1280 A/g_Ir_ at 1.6 V_RHE_. According to the authors, the interactions and chemical bonding between the N‐rich environment and the precious metal nanoparticles modulated the atomic coordination and electronic structure of Ir active sites, promoting OER. XPS measurements showed a 0.2 eV negative shift of Ir4 f photoelectron line in the supported catalyst compared to IrO_2_ while EXAFS experiments exposed a compressed Ir−Ir bond in IrO_2_/GCN compared to the benchmark. A wavelet transformation of the EXAFS spectrum also confirmed the presence of more abundant low‐coordinated Ir sites in the supported nanoparticles. All of these resulted in a modified interaction between Ir and the O‐intermediate of OER in IrO_2_/GCN, promoting a good activity. In addition, GCN nanosheets are superhydrophilic which promoted the adsorption of the electrolyte and desorption of gas during the reaction, according to the authors (Figure 4g). Finally, IrO_2_/GCN presented better stability than the IrO_2_ benchmark under ADT due to the strong bonding between IrO_2_ and the support, inhibiting agglomeration and corrosion of the nanoparticles (Figure 4h). In addition, the IrO_2_/GCN with 40 wt% Ir was placed in a laboratory water splitting device (Pt/C was placed on the cathode) and could sustain for 24 hours at 1.6 V with current retention of 78.5 %.

Therefore, doped‐graphitic carbon support seems to be able to sustain the harsh OER conditions in acidic media, overcoming the biggest carbon problem at this potential, i. e. carbon oxidation. The possibility of using carbon support for iridium, which seems to hinder the CO_2_ formation, could drastically decrease the amount of iridium in a real PEMWE. However, the stability of carbon should still be studied under real conditions in PEMWE, i. e. high current densities, high temperatures, and start‐up/shut‐down cycles, to ensure the long‐term durability of such catalysts.

### 2.2 Metal oxides

Another class of materials, studied as potential supports for OER catalysts are based on transition metal oxides.[^124^, ^125^] The main advantage of such materials is their high thermodynamic stability under oxidative conditions,^[126]^ and the main disadvantage is their insufficient electrical conductivity. Nevertheless, their properties can be chosen or tuned to possess sufficient conductivity.

#### 2.2.1 Tin‐oxides (SnO_2_)

SnO_2_ has a conductivity of 1.82×10^−8^ S/cm,^[127]^ which means that the required conductivity of an electrocatalyst could only be reached if some substance with metallic conductivity, such as iridium, is added. Nevertheless, according to the Pourbaix diagram, Sn passivates into SnO_2_ and is stable at pH 0–12 if the potential is kept above 0.2 V vs RHE,^[128]^ which makes it a widely considered candidate for OER support, despite the low intrinsic conductivity.

**Figure 5:**
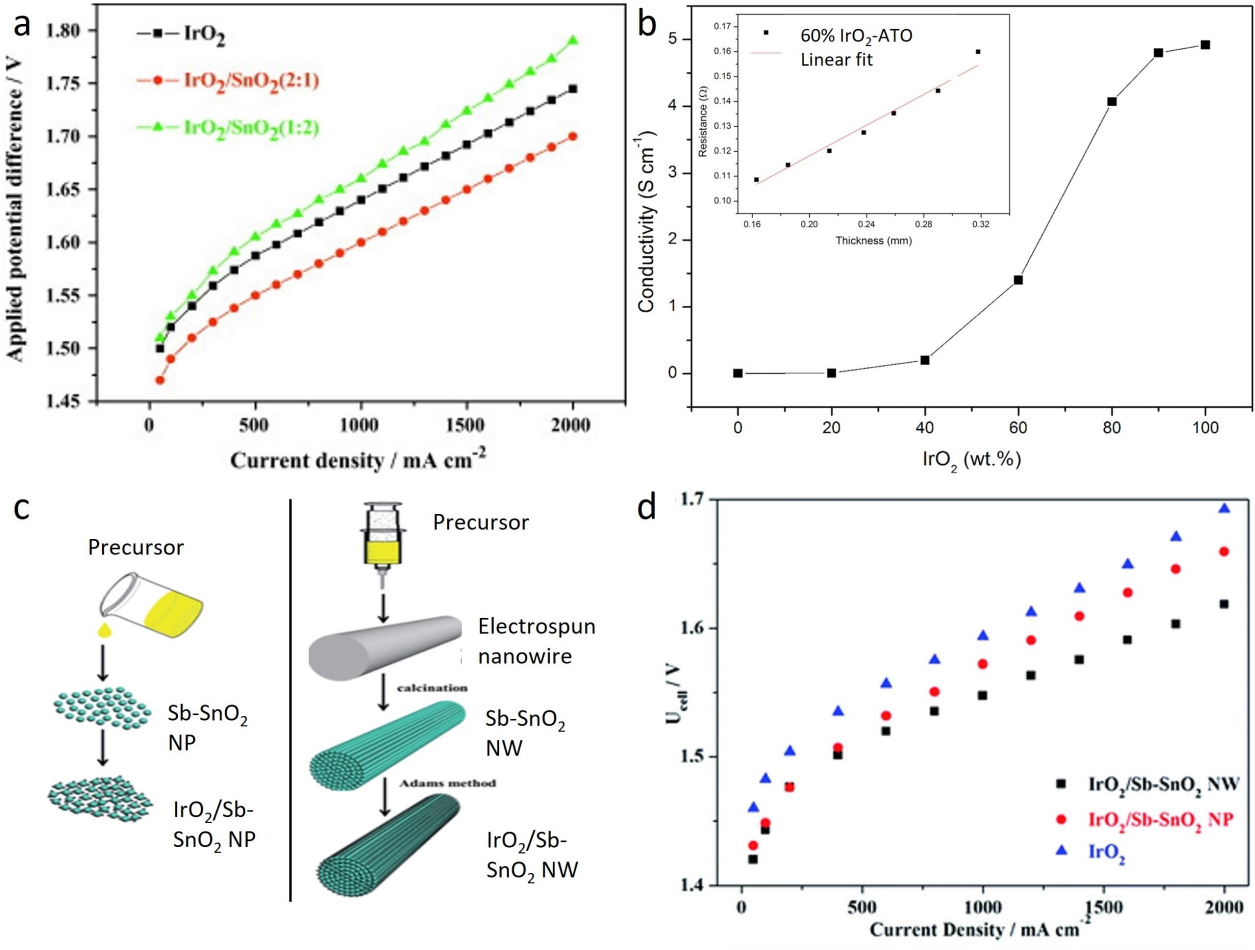
a) Applied potential difference of a solid polymer electrolyte water electrolysis cell at 80 °C. Reproduced with permission.^[129]^ Copyright 2012, Elsevier. b) Conductivity of IrO_2_‐ATO with respect to the IrO_2_ loading. Inset shows the relation between thickness and resistance of a 60 wt% IrO_2_‐ATO film. Reproduced with permission.^[133]^ Copyright 2014, Elsevier. c) Schematic illustration of the synthetic route of IrO_2_/Sb−SnO_2_ NP and IrO_2_/Sb−SnO_2_ NW.^[135]^ d) Current‐voltage curves of PEMWE cells with different anode catalysts at 80 °C. Reproduced with permission.^[135]^ Copyright 2015, The Royal Society of Chemistry.

Xu et al. investigated SnO_2_ as a support and promoting agent for IrO_2_ nanoparticles.^[129]^ The addition of SnO_2_ promoted the dispersion of Ir as expected from a supported catalyst but also increased the removal efficiency of OH‐species. A ratio of 2 : 1 (IrO_2_:SnO_2_) was found to be optimal to enhance activity toward OER (Figure 5a). At higher Sn content the conductivity excessively dropped which inhibited the activity, while a lower amount of Sn was detrimental to the dispersion of IrO_2_.

Even if the stability of SnO_2_ is an advantage for its use as an OER support, its poor electrical property still needs to be improved. In this regard, doping with various elements such as Sb, F, In, Ta or Nb, was used to increase the conductivity of the support. It was found that the intrinsic conductivity of the support is particularly important in single‐cell tests and at high current densities, while it does not influence the half‐cell tests to the same degree.^[130]^

##### 2.2.1.1 Antimony‐doped Tin oxide (ATO)

In order to increase the conductivity of the SnO_2_‐support, several dopants have been investigated. Among them, antimony‐doped tin oxide (ATO) was highlighted as particularly promising by Shao‐Horn's group after screening several oxides as potential supports for OER in acidic media.^[131]^ ATO possesses lower electrical resistance (10^−2^–10^−3^ Ω.cm^[132]^) than pure SnO_2_, high stability at pH 0 and at potentials up to 2 V vs RHE, and a relatively low priced, which is an unavoidable requirement for industrial utilization. Therefore, numerous groups have been evaluating the performances of iridium supported on ATO‐based supports.[^133^, ^134^, ^135^, ^136^, ^137^, ^138^, ^139^, ^140^, ^141^, ^142^] Puthiyapura et al. studied the influence of different loadings of Ir (deposited on commercial ATO nanoparticles, BET 20–40 m^2^/g, conductivity 4.29 .10^−3^ S/cm) on the OER performances.^[133]^ Loading of a minimum of 40 wt% Ir was required to achieve a resistivity lower than 10 Ω.cm (Figure 5b), which was advised by Marshall et al. for suitable anode material in PEMWE.^[143]^ However, at least 60 wt% Ir on commercial ATO was necessary to reach the same conductivity as IrO_2_. Catalysts with more than 60 wt% Ir showed better OER performances compared to IrO_2_ benchmark which were attributed to better dispersion of IrO_2_ and higher electrochemical surface area.

The loading of iridium needed (>60 wt%) is still too high to reach worldwide commercialization and an improvement in intrinsic conductivity of ATO was further researched. Liu et al. synthesized and compared ATO supports with two different morphologies, nanowires (NW, BET 60 m^2^/cm) and nanoparticles (NP, BET 54 m^2^/cm) (Figure 5c).^[135]^ Both homemade supports exhibited higher conductivity than commercial ATO with 0.83 and 0.76 S/cm for NW and NP, respectively. After deposition of Ir nanoparticles, the catalysts (IrO_2_/ATO NW and IrO_2_/ATO NP) presented better OER performances than benchmark IrO_2_ as expected due to the higher number of exposed active sites and different electronic environment as previously shown. Interestingly, IrO_2_/ATO NW exhibited better OER performances than its counterpart deposited on ATO NP, which could not be related to the better intrinsic activity of the active sites (Figure 5d). Therefore, the authors suggested that the improved activity could be related to the geometry of the nanowire support. Indeed, nanowires are more accessible due to an open structure that can better facilitate the mass transport of gaseous products.

**Figure 6:**
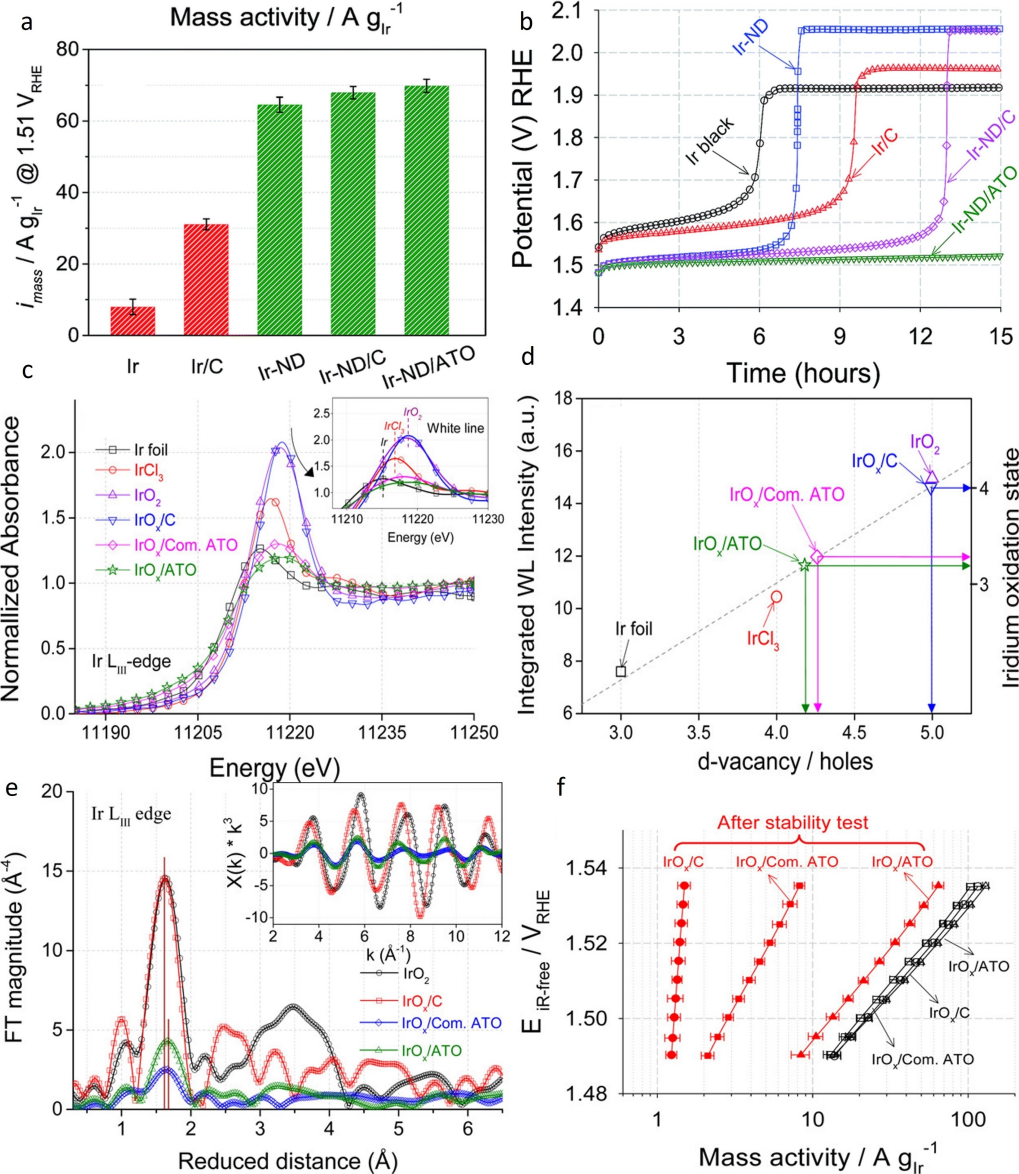
a) Mass activity of Ir‐based catalysts at 1.51 V_RHE_.^[140]^ b) Chronopotentiometry accelerated degradation test (ADT) of Ir‐based catalyst, 10 mA/cm^2^. Reproduced with permission.^[140]^ Open access 2015. c) Normalized Ir L_III_‐edge XANES spectra of Ir‐based catalysts. Inlet shows a magnified region of Ir L_III_‐edge XANES white line.^[138]^ d) Integrated white‐line versus d vacancy and Ir oxidation state of Ir‐based catalysts.^[138]^ e) Fourier transform of EXAFS spectra at the Ir L_III_‐edge collected for Ir‐based catalysts. Inset shows the corresponding k^3^‐weighted EXAFS spectra.^[138]^ f) iR‐corrected Tafel slope of Ir‐based catalysts before (black) and after (red) the stability test. Reproduced with permission.^[138]^ Copyright 2016, American Chemical Society.

Strasser's group and collaborators also work on various ATO modifications to be used as a support for different Ir‐based catalysts.[^138^, ^139^, ^140^, ^144^] They developed tin oxide supports with different dopants (Sb, F and In) with higher surface area (125–263 m^2^/g), high crystallinity and a mesoporous structure.^[144]^ The doped tin oxide materials also showed an enhanced conductivity compared to commercial ATO (0.102–0.295 S/cm vs 4.29.10^−3^ S/cm) and are comparable to the work of Liu et al.^[135]^ Afterwards, these new supports, notably the antimony‐doped one, were used in several studies conducted by the group.[^138^, ^139^, ^140^] IrNiO_x_@Ir core‐shell nanoparticles deposited on the homemade ATO support displayed better activity and stability than when deposited on commercial ATO and on carbon.^[139]^ Similarly, Ir nanodendrites (Ir−ND), while intrinsically more active than Ir black due to their morphology, demonstrated an improved activity when deposited on ATO compared to Ir/C or Ir−ND supported on carbon (Figure 6a).^[140]^ The ATO support also brought enhanced stability by hindering the agglomeration of nanoparticles oppositely to the carbon support studied in this work (Figure 6b). To understand the reason behind the improved performances of the catalysts using ATO, the group conducted an in‐depth study of the interaction between the support and the precious metal nanoparticles using XANES, EXAFS, TEM and depth resolved XPS.^[138]^ This work highlighted a difference in iridium oxidation level after electrochemical activation (cycles from 0.05 to 1.5 V_RHE_) between IrO_x_/ATO, IrO_x_/C and IrO_x_/commercial ATO. The Ir L_III_‐edge in XANES spectra of the samples underlined the difference in d vacancy depending on the support (Figures 6c and 6d). Thus, oxidation levels of 3.2+, 3.3+ and 4+ were identified for IrO_x_/commercial ATO, IrO_x_/ATO and IrO_x_/C, respectively. Afterwards, EXAFS measurements were employed to obtain information on the local structure and Ir coordination level. The authors found that Ir−O distances were shifted to larger values in IrO_x_ supported on ATO (homemade and commercial), accordingly to a lower average oxidation number (Figure 6e). Finally, the depth‐resolved XPS Ir 4 f spectra revealed the contribution of small Ir^3+^ and metallic Ir for the ATO‐support samples, corroborating the lower oxidation state found with EXAFS and XANES. Therefore, the authors ascribed the improved performances, activity and stability wise (Figure 6f), of ATO‐supported Ir nanoparticles over Ir/C and IrO_2_ to the stronger metal/metal‐oxide support interaction which influenced the oxidation state of the active iridium, the Ir oxide layer thickness and its surface morphology, all important parameters that govern stability and activity.

**Figure 7:**
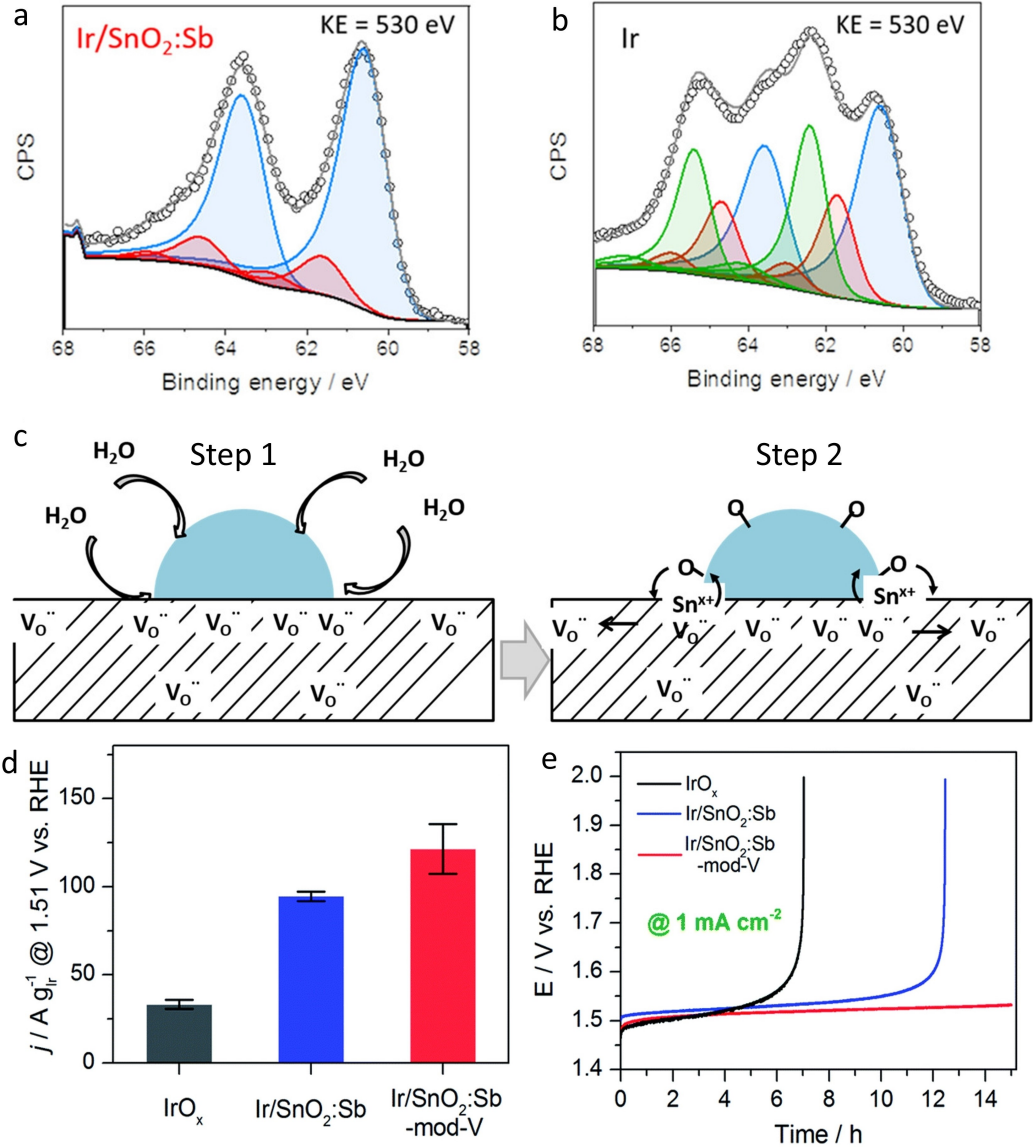
a) XPS spectra at the kinetic energy of 530 eV of IrO_2_/SnO_2_:Sb after potential cycling^[141]^ and b) XPS spectra at the kinetic energy of 530 eV of unsupported Ir after potential cycling.^[141]^ Color code: metallic Ir, blue; Ir(IV) and its satellite, red; and Ir(III) and its satellite, green. c) Schematic representation of the oxygen spill‐over to the SnO_2_:Sb support under anodic polarization. Step 1: electrochemisorption and dissociation of water on the surface of Ir. Step 2: adsorbed oxygen spill‐over from Ir to the SnO_2_:Sb support resulted in partial oxygen vacancy refilling. Reproduced with permission,^[141]^ Copyright 2020, American Chemical Society. d) Mass activity of Ir‐based catalysts at 1.51 V_RHE_ ^[145]^ e) Chronopotentiometry ADT of Ir‐based catalysts, 1 mA/cm^2^.^[145]^ Open access, 2017.

This hypothesis was later confirmed by Saveleva et al. using operando near ambient XPS experiments.^[141]^ Here, the authors studied the mechanism behind the improved activity of Ir particles supported on ATO by applying near ambient pressure XPS (NAP‐XPS) on Ir/ATO catalyst integrated into a membrane electrode assembly. Again, the in‐situ experiment revealed a higher presence of metallic Ir in the Ir4 f spectrum despite the oxidative protocol compared to unsupported catalysts. This corresponds to an estimated oxide layer of 0.3–0.4 nm (under OER conditions) for Ir/ATO while unsupported Ir nanoparticles have an oxide layer of 0.6–0.7 nm. In addition, the presence of Ir(III) was found for unsupported iridium but not for Ir/ATO (Figure 7a and 7b). Ir(III) was suggested to be involved in the degradation mechanism and its absence could explain the difference in the observed stability.^[70]^ Moreover, the authors suggested an oxygen spill‐over from Ir to the Sb‐doped SnO_2_ support from NAP‐XPS results (Figure 7c).^[141]^ Indeed, the Sn 3d_5/2_ binding energy (487.3±0.2 eV) was systematically decreasing with increasing electrode potential when used as a support for Ir, but not when used alone. This reduced binding energy with potential was attributed to the filling of oxygen vacancy due to spill‐over from Ir nanoparticles.

Hartig‐Weiss et al. managed to outperform all previously published catalysts with ATO support by synthesizing ATO material with one order of magnitude higher conductivity (2 S/cm) and still sufficient surface area (50 m^2^/g).^[142]^ This new material allows extra‐low loadings of Ir (11 wt%) while reaching the impressive mass activity of 1100 A/g_Ir_ at 1.45 V_RHE_ (at 80 °C). In accordance with previous studies, the iridium nanoparticles were not fully oxidized to 4+ state after the electrochemical protocol, which enhanced their intrinsic activity. The latter and high dispersion of small nanoparticles (1.5–1.8 nm, theoretical size of 3.2 nm after oxidation) and a strong SMSI are likely responsible for the high activity observed. However, no stability test was performed and the presence of Ir(III) in the XPS spectra after oxidation brings the question of the stability of the catalyst in the long term.

Finally, the ATO can be further modified by the addition of more elements.[^145^, ^146^, ^147^] For example, Wang et al. added vanadium during the synthesis of the catalyst (Ir/SnO_2_:Sb‐mod‐V).^[145]^ The vanadium additive is not active for OER and did not influence the conductivity of the support but superior performances were still observed over its Ir/SnO_2_:Sb counterpart and IrO_x_ benchmark. An OER mass activity of 121.5 A/g_Ir_, 94.6 A/g_Ir_ and 33.6 A/g_Ir_ were measured at 1.51 V_RHE_ for Ir/SnO_2_:Sb‐mod‐V, Ir/SnO_2_:Sb and IrO_x_, respectively (Figure 7d). In addition, Ir/SnO_2_:Sb‐mod‐V could sustain 15 hours at a current density of 1 mA/cm^2^ while Ir/SnO_2_:Sb and IrO_x_ were degraded after approximatively 7 and 12 h, respectively (Figure 7e). The authors assigned better performances to geometric effects. Indeed, the presence of V in the sample allowed a highly porous and stable structure as well as increased roughness. The 3D porous structure boosts the Ir utilization and the electrochemical surface area, as measured by Cu‐under potential deposition.

##### 2.2.1.2 Other dopants

**Figure 8:**
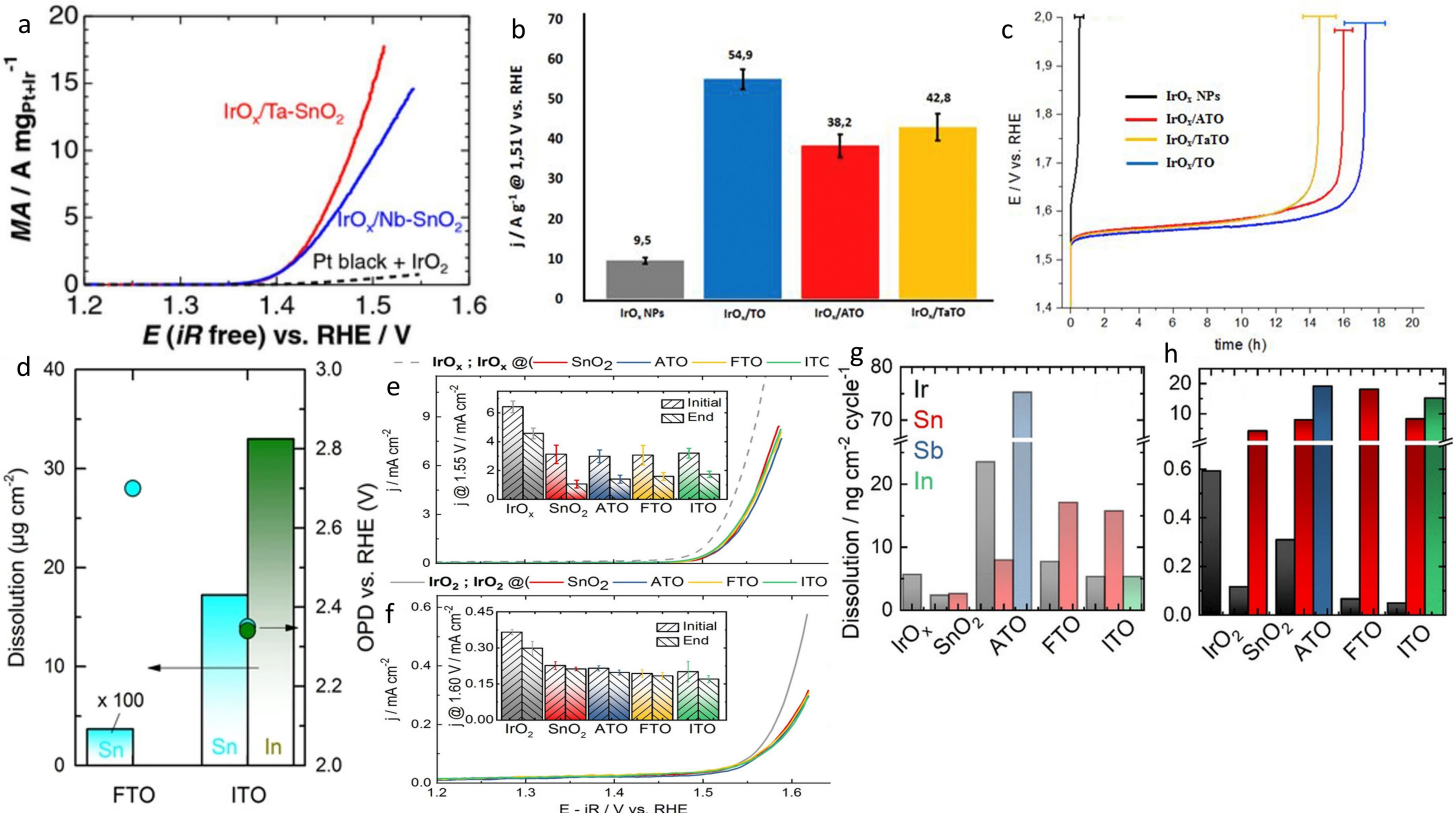
a) iR‐free anodic polarization curve for IrO_x_/Nb−SnO_2_, IrO_x_/Ta−SnO_2_ and conventional (IrO_2_+Pt) catalysts in 0.1 M HClO_4_ solution at 80 °C.^[150]^ Open access 2017. b) Mass activity of Ir‐based catalysts at 1.51 V_RHE_.^[151]^ c) Chronopotentiometry ADT of Ir‐based catalysts at 1 mA/cm^2^. Reproduced with permission.^[151]^ Copyright 2019, Elsevier d) Comparison of the dissolution during OER, normalized by the portion of the respective element in the material (bars) and onset potential of dissolution (OPD, bullets).^[154]^ Open access 2017. e) iR‐corrected polarization curve of IrO_x_‐based catalysts. Inset: activity at 1.55 V_RHE_ before and after 1000 cycles between 1.1 and 1.6 V_RHE_.^[155]^ f) iR‐corrected polarization curve of IrO_2_‐based catalysts. Inset: activity at 1.6 V_RHE_ before and after 1000 cycles between 1.1 and 1.6 V_RHE_.^[155]^ g) Dissolved amount of IrO_x_ in IrO_x_‐based catalysts during three cycles between 1.1 and 1.6 V_RHE_ ^[155]^ and h) Dissolved amount of IrO_2_ in IrO_2_‐based catalysts during three cycles between 1.1 and 1.6 V_RHE_.^[155]^ Open access 2020.

In addition to Sb, other elements have also shown a positive effect on the overall OER performances, like In, Nb, Ta or F, albeit with a lower conductivity compared to ATO.[^144^, ^148^, ^149^, ^150^, ^151^, ^152^, ^153^] For example, Nb and Ta‐doped SnO_2_ possess an order of magnitude lower conductivity than ATO, but remarkable activities were still measured for OER.^[149]^ Ohno et al. synthesized IrO_x_ on Nb−SnO_2_ and Ta−SnO_2_ by flame pyrolysis.^[150]^ A mass activity of 10 and 15 A/mg_Ir_ at 1.5 V vs RHE, was recorded for IrO_x_/Nb−SnO_2_ (11.3 Ir wt %) and IrO_x_/Ta−SnO_2_ (10.3 Ir wt %), respectively. This corresponded to 21 and 32 times higher than the mass activity (0.44 A/mg_Ir+Pt_) of a conventional Ir+Pt catalyst used in URFC (Figure 8a). Such improved activity was ascribed to the higher surface of supported 2 nm iridium nanoparticles compared to the 10 nm unsupported benchmark. In addition, a lower Tafel slope was observed for the supported catalyst suggesting a promoted OER and thus an interaction between IrO_x_ nanoparticles and the support. Moreover, IrO_x_/Nb−SnO_2_ could sustain a constant current of 4 A/mg_Ir_ at 80 °C for more than 50 hours.

The positive effect of Ta‐doping was challenged by Sola‐Hernandez et al. The authors compared IrO_x_ (30 wt%) on Ta−SnO_2_, ATO and SnO_2_ supports as well as unsupported IrO_x_ in simulated PEMWE conditions.^[151]^ The Ta‐doping resulted in lower conductivity of the support than Sb‐doping, i. e.17 10^−4^ vs 8.2 10^−2^ S/cm, respectively. Nonetheless, similar OER performances were recorded, with both materials surpassing unsupported IrO_x_. However, IrO_x_/SnO_2_ was found to present the best mass activity, 54.9 A/g at 1.51 V_RHE_ compared to IrO_x_/TaTO (42.8 A/g) and IrO_x_/ATO (38.2 A/g) (Figure 8b). The authors explained this surprising result by the very thin film that is used in a standard rotating disk electrode (RDE) set‐up where the intrinsic conductivity of the support does not play a role, oppositely to the PEMWE devices where films are 150–200 times thicker. In addition, the stability of the doped catalysts was also found to be worse than the stability of Ir/SnO_2_ during chronoamperometry at 1 mA/cm^2^ (Figure 8c). This highlights one very important discrepancy between RDE experiments and real devices which is commonly ignored by the materials science community.

Fluorine^[148]^ and Indium^[152]^ are some of the other studied doping elements which lead to satisfying results. Ledendecker et al. used liquid atomic layer deposition synthesis to deposit various amounts of Ir‐oxide layer on FTO and ITO support.^[153]^ The authors observed that the specific activity of IrO_x_/FTO was lower than IrO_x_/ITO while both surpassed the IrO_2_ benchmark. In addition, the authors stated that the specific and mass activities are highly susceptible to the support material as the best activities were recorded after 5 layers of Ir on ITO but 20 layers on FTO, with 35 A/mg_Ir_ and 2.5 A/mg_Ir_ at 1.65 V_RHE_, respectively. However, the opposite was observed for the durability of the catalysts where IrO_x_/FTO was more stable than IrO_x_/ITO. The authors explained it by the difference in IrO_x_ crystallinity, which is mostly amorphous for the non‐stable IrO_x_/ITO and mostly crystalline in IrO_x_/FTO.

The doping of stable SnO_2_ support seems a good strategy to improve the activity of the IrO_x_ nanoparticles and their utilization. However, the stability of such doped supports is still not clear. In this context, Geiger et al. studied the stability of ATO, ITO and FTO with a scanning flow cell coupled to an inductively coupled plasma mass spectrometer (SFC‐ICP‐MS).^[154]^ The Sn dissolution in ATO, FTO and ITO was recorded under the operating potential window. The latter showed a two order of magnitude increased dissolution of Sn, indicating a destabilization of Sn in In−SnO_2_. The explanation advanced by the authors is that the intensive dissolution of In leaves some under‐coordinated Sn atoms which are then prone to dissolve. Indeed, In heavily dissolves in acidic media, and 10 nm of the film could be lost in just 12 h under OCP conditions (Figure 8d).^[154]^ This could induce a loss of conductivity and further decrease the catalyst performance, and therefore the utilization of such support was not recommended by the authors. On the other hand, Sb and Sn are relatively stable in the potential window of 0.36 V_RHE_<E<1.1 V_RHE_ and −0.29 V_RHE_<E<1.45 V_RHE_, respectively. Therefore, it is likely that a SnO_2_‐rich layer is formed on the surface, which once again limits the conductivity of the support. Thus, the authors emphasized the importance of controlled synthesis and the quality of film for the stability and conductivity of the ATO support. Finally, FTO shows the best stability probably due to the replacement of oxygen by F atoms instead of cation exchange. However, it exhibits the lowest conductivity. Together with the conclusion of the previous study, showing the lowest activity improvement, this support is thus not recognized as a promising material for application in OER.^[144]^

In a follow‐up study, the same group questioned the use of dopants as they found similar activity for Ir on Sn‐based supports with and without dopants (Figure 8e).^[155]^ They investigated the conductivity, activity, and stability of unsupported IrO_x_ and IrO_x_ on ATO, FTO and ITO. The samples were also calcinated to form IrO_2_ and the same study was conducted on these four new samples (Figure 8f). First, the increased conductivity of the support after doping shown in previous studies was confirmed by the authors, with a 20‐fold improvement for ATO over SnO_2_, with 1.1 10^−1^ S/cm and 5.9 10^−3^ S/cm, respectively while the conductivity of FTO and ITO were measured as 4.9 10^−2^ S/cm and 2.0 10^−2^ S/cm, respectively. Nevertheless, the difference in conductivity was minimized after the deposition of IrO_x_ on the support, with 7.0 10^−2^, 1.5 10^−1^, 1.0 10^−1^ and 8.0 10^−2^ S/cm for SnO_2_, ATO, FTO and ITO, respectively. Similar trend was observed after calcination. The electrochemical tests did not reveal any significant difference in OER activity between IrO_x_ deposited on the various supports, however all catalysts showed lower activity compared to the unsupported IrO_x_ with 41.3 mA/mg_Ir_ for supported catalysts against 85.7 mA/mg_Ir_ without support at 1.55 V_RHE_. The same trend was observed after calcination and formation of IrO_2_ from IrO_x_. The authors explained this difference from previous studies by suggesting an investigation into the effect of Ir loading on the catalytic performances.^[155]^ In terms of stability, unsupported IrO_x_ lost around 30 % of activity after 1000 cycles between 1.1 and 1.6 V vs RHE while the activity of IrO_x_/ATO, IrO_x_/FTO and IrO_x_/ITO decreased by around 50 %. The IrO_x_/SnO_2_ was found to be the least stable with a loss of around 65 % of its activity. After calcination, the unsupported IrO_2_ was the least stable with 20 % loss of activity while all the supported catalysts maintained around 90 % of activity in accordance with the higher stability of crystalline Ir‐oxide over amorphous ones. An online dissolution analysis experiment was then conducted to record the dissolution of different metals during operation (Figure 8g and 8 h). In accordance with stability test, Ir dissolved less after calcination, confirming the higher stability of IrO_2_ over IrO_x_. Interestingly, Sn is more prone to dissolution in the doped support for both IrO_x_ and IrO_2_ containing catalysts. Therefore, the doping of the support negatively impacts its stability, due to the dissolution of the doping element, especially for In and Sb. Moreover, the dissolution of Ir for IrO_x_ is in the order SnO_2_<ITO=unsupported<FTO<ATO and for IrO_2_ (calcinated materials), the trend is ITO=FTO=SnO_2_<unsupported<ATO. Hence, doping the support is not always beneficial for the stability of Ir, especially if the samples are not annealed. The two studies conducted by Cherevko's group show that the doping of SnO_2_ support could not be necessarily beneficial for the stability of the catalyst and further questioned the use of such supports in PEMWE.[^154^, ^155^] This conclusion agrees with Sola‐Hernandez study^[151]^ but not with other groups.[^138^, ^141^, ^149^, ^153^] Thus, it is primordial to develop more stable doped‐SnO_2_ materials for future utilization in PEMWE and to conduct more work to understand the discrepancy between the different results. They could be related to the various and disparate parameters used during the testing of the materials.

#### 2.2.2 Titanium‐oxide (TiO_x_)

Another promising class of supports for OER is based on titanium oxide.[^79^, ^125^, ^156^, ^157^, ^158^, ^159^, ^160^, ^161^] Indeed, TiO_2_ is known to be stable under OER conditions. However, it is a n‐type semiconductor and therefore not sufficiently electronically conductive for practical, high‐current electrocatalytic applications. Thus, the intrinsic conductivity of Ti‐based support needs to be adjusted. To address this issue, several possibilities have been employed such as higher loading of Ir,^[148]^ doping of TiO_2_[^162^, ^163^, ^164^] or use of its suboxides.[^165^, ^166^, ^167^]

##### 2.2.2.1 TiO_2_

Titanium dioxide is particularly stable under acidic conditions and oxidative environment, but its conductivity is limiting its potential usage as electrocatalyst support. Nonetheless, sufficient coverage of iridium on the support can bring the necessary conductivity and still lower usage of this precious metals.[^125^, ^148^, ^157^, ^158^] A loading of 60 wt% of Ir was found to increase the conductivity to the same level as IrO_2_ on ITO.^[148]^ Counterintuitively, using TiO_2_ support with a lower surface area could boost the activity as stated by Mazur et al.^[157]^ They studied IrO_2_ on three different non‐conductive TiO_2_ supports with different surface area, and they kept a constant weight ratio IrO_2_/TiO_2_ of 0.6. When the surface area of the support was too high, a conductive film could not form on top, thus limiting the electrocatalytic reaction. On the other hand, the IrO_2_ deposited on the TiO_2_ with the lower specific surface area (10 m^2^/g) formed an interconnected network and the overall catalyst reached conductivity close to the unsupported IrO_2_. This highlights the importance of reaching sufficient conductivity while using TiO_2_ as support. In addition to the surface area of the support, its crystallinity could play a role in its interaction with the active precious metal. Fuentes et al. conducted a preliminary study of multimetallic electrocatalysts, mixed of Pt, Ru and Ir, on anatase and rutile TiO_2_.^[156]^ The activity of Pt : Ru/TiO_2_ towards OER was 44 % higher with anatase support than with the rutile counterpart, consistently with a similar study for the methanol oxidation,^[168]^ but no explanation was provided by the authors. Both supported catalysts showed better activity than the unsupported catalyst which was attributed to the higher surface area due to dispersed nanoparticles.

**Figure 9:**
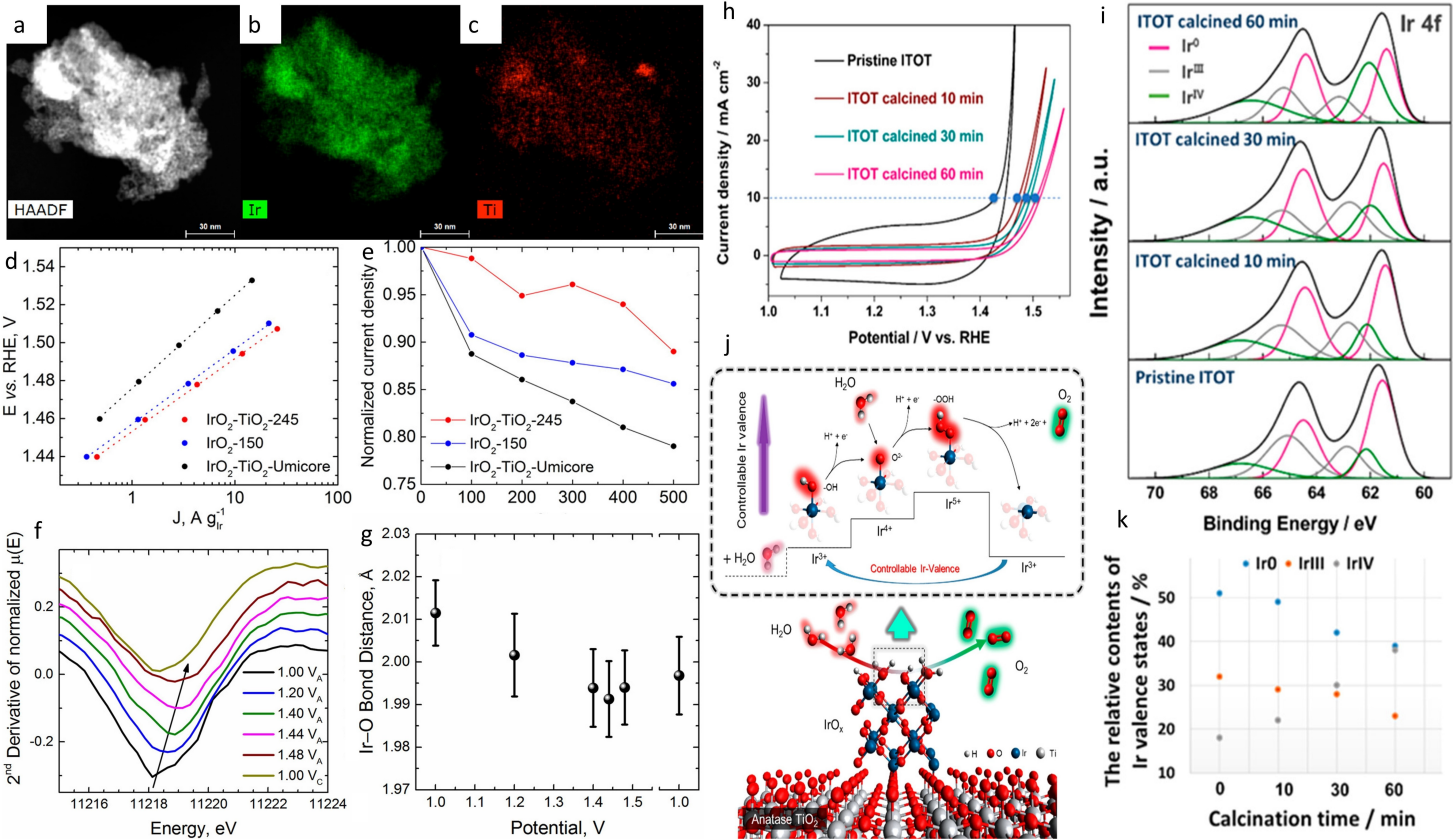
a) HAADF‐STEM image of IrO_2_−TiO2–245.^[125]^ b) Iridium and c) Ti EDX maps of IrO_2_−TiO_2–_245^[125]^ d) Tafel slope of Ir‐based catalysts.^[125]^ e) Stability of Ir‐based catalysts.^[125]^ f) Second derivative of in‐situ XANES adsorption edge of IrO_2_−TiO_2–_245 as a function of the applied potential.^[125]^ g) EXAFS‐determined Ir−O bond distance as a function of the applied potential. Reproduced with permission.^[125]^ Copyright 2017, American Chemical Society. h) Polarization curves of ITOT catalysts after calcination at 350 °C for different times.^[159]^ i) Ir4 f XPS spectra of the ITOT catalysts.^[159]^ j) Proposed OER mechanism for the ITOT catalysts.^[159]^ k) Relative contents of Ir valence states in the ITOT catalysts. Reproduced with permission.^[159]^ Copyright 2019, American Chemical Society.

A few years ago, Schmidt's group presented an active, stable and high surface area IrO_2_/TiO_2_ electrocatalyst with 40 mol% of Ir.^[125]^ Their one‐step synthesized material possessed a surface area of 245 m^2^/g, higher than pure IrO_2_ synthesized with the same methodology (150 m^2^/g) and benchmark IrO_2_−TiO_2_ from Umicore (34 m^2^/g). The overall conductivity of the material was 0.26 S/cm and was mostly due to some iridium‐rich regions as seen by EDXS (Figure 9a–c). The XRD results showed the presence of rutile IrO_2_ and both rutile and anatase TiO_2_ in the material. Therefore no differentiation based on the crystalline form of the TiO_2_ could be concluded. Nonetheless, the same synthesis for only TiO_2_ only leads to anatase, which hints at an interaction between IrO_2_ and TiO_2_ resulting in the rutile structure. In terms of electrochemical performances, the homemade catalyst was more active than both unsupported synthesized IrO_2_ and benchmark IrO_2_−TiO_2_‐Umicore with a mass activity of 70 A/g_Ir_, 44 A/g_Ir_ and 14 A/g_Ir_ at 1.525 V_RHE_, respectively (Figure 9d). The authors ascribed the better performances of both homemade catalysts (supported on TiO_2_ and unsupported) to the presence of surface iridium hydroxo species (*OH) as indicated by XANES and EXAFS spectra. Indeed, the benchmark catalyst had a higher adsorption edge energy in XANES spectra, suggesting a higher oxidation state of Ir centre and thus less Ir3+ compared to the synthesized materials. At the same time, the fitting of EXAFS showed that the Ir−O bond in IrO_2_−TiO_2–_245 (homemade) and unsupported IrO_2_ is longer, indicating the presence of Ir3+ and thus lower oxidation state compared to the Ir4+ of IrO_2_−TiO_2_‐Umicore. Furthermore, similar Tafel slopes were found for IrO_2_−TiO_2–_245 and the unsupported IrO_2_ suggesting that TiO_2_ does not influence the reaction pathway. Afterwards, the authors conducted a potential stepwise degradation, namely 500 cycles from 1 to 1.6 V_RHE_ with holding 10 s at each potential, to study the stability of the catalysts. None of the samples was immune to the degradation protocol but higher stability of IrO_2_−TiO_2–_245 was observed, followed by IrO_2_ and IrO_2_−TiO_2_‐Umicore, which was the least stable (Figure 9e). The improved stability was attributed to the particle‐support interaction, already suggested by the presence of rutile TiO_2_. Moreover, operando XAS study showed a shortening of the Ir−O bond with increasing applied potential indicating the transition from a mixed Ir3+/4+ to a mostly Ir4+ population for IrO_2_−TiO_2–_245 (Figure 9f and 9 g). However, oppositely to the previous report for unsupported catalysts, this transition was not fully reversible.^[76]^ Thus, TiO_2_ stabilized higher iridium oxidation state and, as lower iridium oxidation states have been associated with lower stability,^[70]^ increased the stability of the catalyst.

Similar conclusions were drawn by Cheng et al. in their work on IrO_x_−TiO_2_−Ti (ITOT) catalyst.^[159]^ Their synthesized catalyst showed impressive OER performances needing only a potential of 1.43 V_RHE_ to reach a current density of 10 mA/cm^2^ (Figure 9h) and being able to withstand this current density for 100 hours or 700 cycles. The high activity was correlated to the high concentration of OH species recorded by XPS. On the other hand, the outstanding stability was ascribed to the stable content of Ir(0), Ir(III) and Ir(IV) in the catalyst (Figure 9i and 9k). Based on the in‐situ XANES study, EXAFS experiments and DFT calculation, the authors proposed an OER mechanism where the TiO_2_ support contributes to the formation and regeneration of Ir(III) (Figure 9j).

Recently, another approach that combined low iridium loading while limiting the impact of TiO_2_ low conductivity has been proposed by Pham et al., i. e. the synthesis of TiO_2_ core‐shell coated with IrO_2_.^[169]^ Interestingly, the catalyst was tested in a real membrane exchange assembly (MEA) device where it outperformed most of the catalysts and benchmarks, proposed in the literature. Therefore, industrial applications of Ir‐low loadings (0.4 mg_Ir_/cm^2^) on a TiO_2_‐based support seem promising even if, as pointed out by the authors, the lower stability compared to commercial IrO_2_/TiO_2_ was observed.

##### 2.2.2.2 Titanium suboxides (Ti_n_O_2‐n_)

Titanium dioxide is not conductive, but this is not the case with titanium suboxides which contain conductive Ti(III),^[165]^ the most famous one being Ebonex (commercial mixture of Ti_4_O_7_ and some other phases). For that reason, several groups have investigated various titanium suboxides as potential supports for the OER in acidic media.[^166^, ^167^, ^170^, ^171^, ^172^]

**Figure 10:**
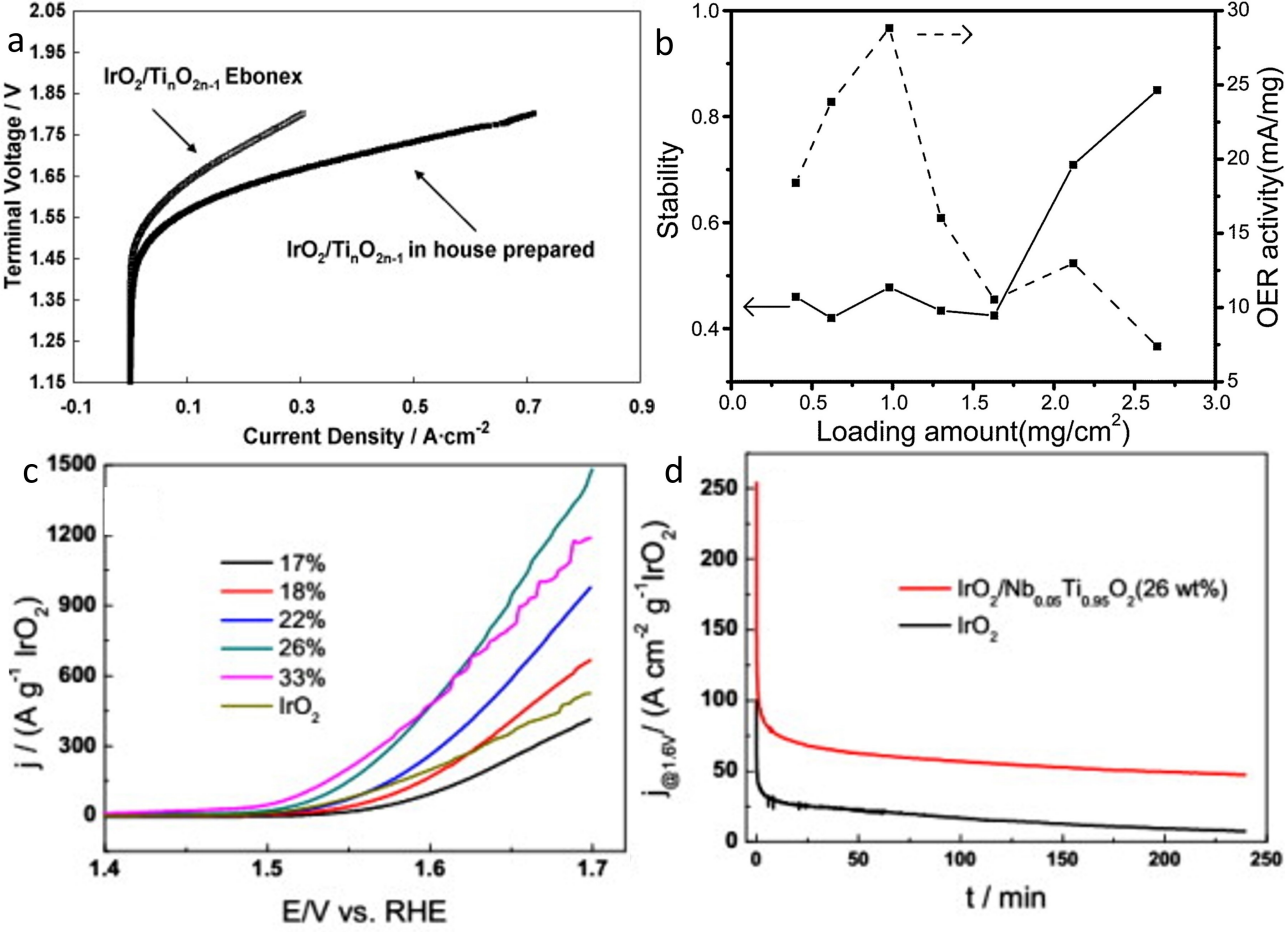
a) MEA polarization curves of IrO_2_/Ti_*n*_O_2*n*–1_ (in‐house) and IrO_2_/Ebonex^®^ at 1 bar abs and 80 °C. Reproduced with permission.^[167]^ Copyright 2009, Elsevier b) Relationship between loading amount and stability (solid line) and loading amount and mass activity after degradation (dashed line). Reproduced with permission.^[172]^ Copyright 2017, Elsevier. c) Mass‐normalized polarization curves of Ir‐based catalysts with various Ir weight loadings.^[162]^ d) Chronoamperometry degradation tests of Ir‐based catalysts, at 1.6 V_RHE_. Reproduced with permission.^[162]^ Copyright 2014, Elsevier.

Twenty years ago, Chen et al. investigated conductive Ti‐based material as potential support for bifunctional catalyst in URFC.^[170]^ Thereby, they synthesized catalysts with various PGMs metals (Pt, Ir, Ru, Os and Ru) on Ebonex, Ti_4_O_7_ and Ti_0.9_Nb_0.1_O_2_. Ebonex and Ti_4_O_7_ were found to not maintain sufficient conductivity for a long period of time under OER conditions due to oxidation, limiting the overall stability of the catalyst. Oppositely, catalysts using the Ti_0.9_Nb_0.1_O_2_ support were stable under OER conditions and will be in detail discussed in the following section. Interestingly, the negative preliminary results from Chen et al. did not stop the exploration of possible stable and conductive Ti‐suboxides. Arico et al. studied IrO_2_ on Ebonex and Ti_n_O_2n–1_ in a real solid polymer electrolyte electrolyzer device and obtained promising activity (Figure 10a) and days long stability.^[167]^ Unfortunately, no explanation for the discrepancy was given by the authors. Nevertheless, it might be speculated that the iridium coverage of the support (30 wt%) was high enough to protect the Ti‐suboxides from oxidation. Indeed, Lu et al. showed that increased coverage of IrO_2_ on a hydrogenated TiO_2_ nanotubes support (with reduced Ti3+) was crucial for the stability of the catalyst and its conductivity (Figure 10b).^[172]^

Therefore, Ti‐suboxides might not be suitable as OER supports with low iridium loadings. Nevertheless, they could be potentially interesting for the next generation of supports for bifunctional catalysts, with promising results being reported for the PtIr/Ti_4_O_7_ catalyst, showing improved stability compared to carbon‐supported catalysts with similar conductivity and activity.^[171]^

##### 2.2.2.3 Doped‐Titanium oxide

TiO_2_ still requires significant Ir loading to reach good conductivity while Ti‐suboxides do not seem to be stable enough under OER conditions due to their oxidation to the non‐conductive TiO_2_. Therefore, the same approach as for tin‐oxide was applied and various dopings of the titanium dioxide were studied.[^162^, ^163^, ^164^, ^173^, ^174^, ^175^, ^176^] Doped TiO_2_ support has already been studied for PEMFC application and porous and conductive materials could be obtained.[^177^, ^178^, ^179^, ^180^, ^181^] Following the promising results from Chen et al. on IrO_2_/Ti_0.9_Nb_0.1_O_2_,^[170]^ Hu et al. synthesized Nb_0.5_Ti_0.95_O_2_ support (83 m^2^/g) with various loadings of IrO_2_ (16–33 wt%).^[162]^ The mass activity of IrO_2_ 26 wt%/Nb_0.05_Ti_0.95_O_2_ was 2.4 times better than that of unsupported IrO_2_ with significantly improved stability, ascribed to the strong anchoring of IrO_2_ nanoparticles on the support (Figure 10c and 10d). Considering the good results obtained so far with low doping of Nb, Hao et al. investigated the effect of doping amount (5, 10 and 20 at%) on the structure, the morphology, and the electrochemical performances of the catalysts.^[173]^ Interestingly, the Nb presence in the support induces the lone formation of anatase TiO_2_, with no rutile peak observed in XRD which should, according to the preliminary study of Fuentes,^[156]^ positively influence the OER activity. In addition, BET measurements were twice higher when Nb was present – independently of the at % – compared to pure TiO_2_ and the average pore diameter was twice lower. The porous morphology of doped materials contributes to their high surface area and leads to a better dispersion of IrO_2_ nanoparticles. The conductivity of the support was also greatly improved by the Nb‐doping with a conductivity of 6.36 10^−1^ S/cm for IrO_2_/Nb 20 at%‐TiO_2_ against 1.13 10^−2^ S/cm for IrO_2_/TiO_2_. In addition, XPS measurements indicated the presence of reduced Ti on the surface and Nb(IV)/Nb(V) redox couple. The latter is capable of forming/breaking Nb−O and Nb‐OH bonds and, according to the authors, it could help the removal of oxygenated species from nearby IrO_2_ and thus increase the turnover frequency of the active sites. Moreover, less Ir(VI) species compared to unsupported IrO_2_ and IrO_2_ on TiO_2_ were observed on the doped‐support. Ir(VI) is particularly important for stability as it corrodes under OER conditions and is a major part of catalyst degradation.^[70]^ The electrochemical performances agree with these hypotheses as the IrO_2_/Nb 20 at% ‐ TiO_2_ was found to be the most active as well as the most stable catalyst.

The same group conducted a similar study with vanadium doping instead of Nb with similar conclusions.^[174]^ Indeed, the presence of V in the porous – mostly anatase – support leads to an increase in specific surface area of the support as well as smaller pores. Again, XPS revealed the presence of V(IV)/V(V) redox couples which helps in the removal of oxygenated species and increases the activity of IrO_2_. However, doping of 30 at% was found to be too high as V_2_O_5_ species were formed and subsequently destabilized the catalyst as they corrode under OER conditions.

The same results were found for IrO_2_ on Ta‐doped TiO_2_ support.^[163]^ In conclusion, introducing a dopant into TiO_2_ provides several advantages in the morphology of the support, i. e. higher surface area and smaller pores, boost the conductivity and positively influence the IrO_2_ nanoparticles′ electrochemical performances. Nonetheless, in our opinion, an online‐ICP‐MS study should be conducted to monitor the possible dissolution of the doping element to assure their long‐term stability as was performed for doped‐TO supports.

##### 2.2.2.4 Other supports based on TiO_2_

**Figure 11:**
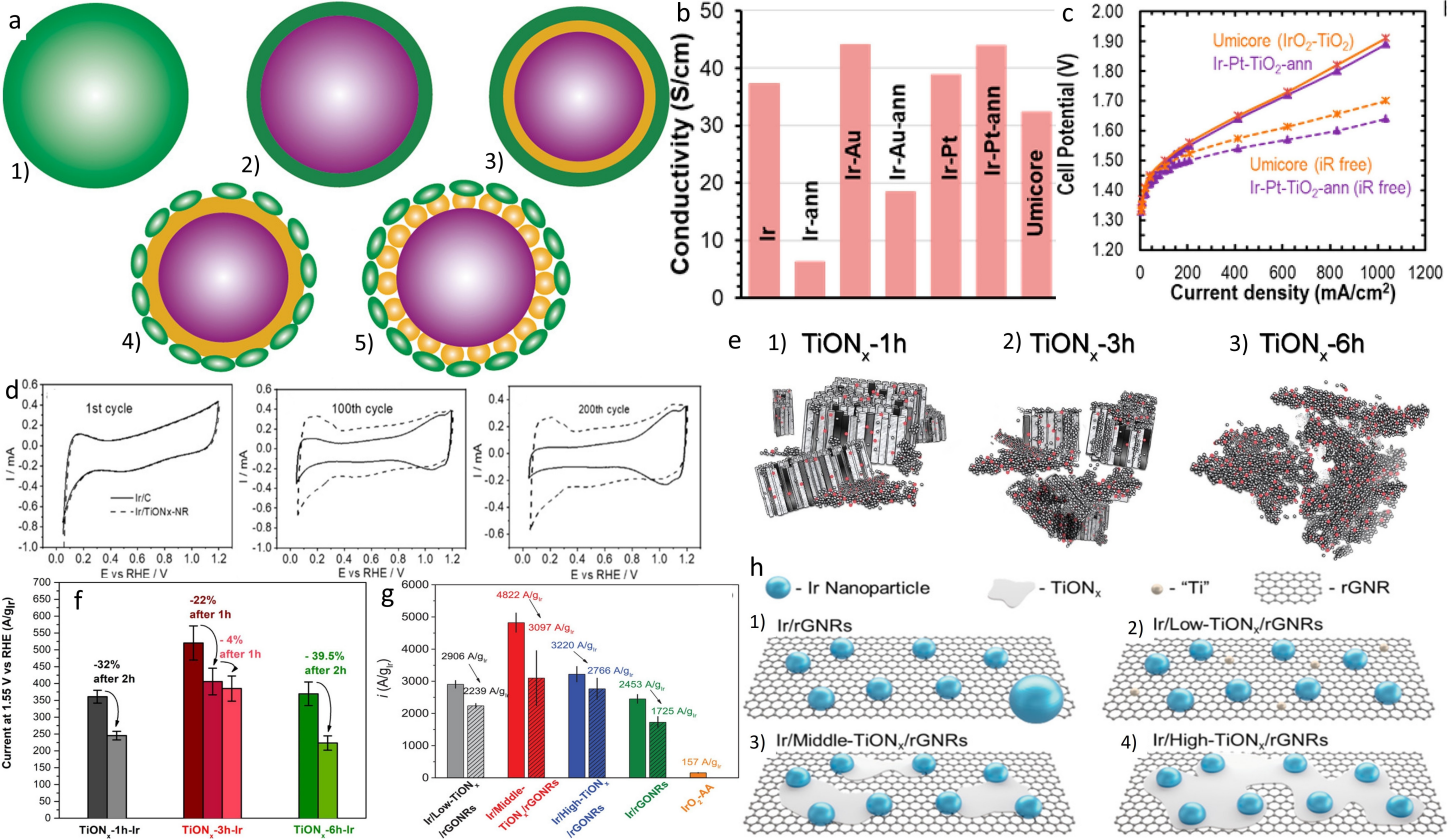
a) Schematic of: 1) bulk Ir catalyst, 2) Ir‐shell coated TiO_2_, 3) proposed catalyst with a Pt or Au intermediate layer, 4) nanostructuring strategy to reduce Ir loading without loss of surface area and 5) nanostructuring of both Ir layer and Pt/Au layer to lower the PGM.^[182]^ b) Conductivity of Ir‐based catalysts, with and without the intermediate layer. ‐ann represents thermally annealed materials.^[182]^ c) Electrolyzer polarization curves of Ir‐based catalysts, 1.0 mg/cm^2^ PGM at the anode. Reproduced with permission.^[182]^ Copyright 2020, American Chemical Society. d) Different cycles of the potential treatment of Ir/C and Ir/TiO_x_N_y_ to highlight the SMSI. Reproduced with permission.^[183]^ Copyright 2019, Wiley‐VCH. e) Schematic of Ir−nh−TiO_x_N_y_ catalysts with support anodized for different times.^[185]^ f) Mass activity at 1.55 V_RHE_ before and after a chronopotentiometry degradation test at 0.1 A/mg_Ir_ of Ir−nh−TiO_x_N_y_ catalysts.^[185]^ Open access 2020. g) Mass activity at 1.55 V_RHE_ before and after a chronopotentiometry degradation test at 2 mA/cm_geo_^2^ of Ir‐based catalysts.^[186]^ h) Schematic of Ir deposited on TiO_x_N_y_−rGNRs supports.^[186]^ Open access 2021.

High iridium loadings and doping are not the only possible strategies to improve the conductivity of TiO_2_ without sacrificing stability. For example, Danilovic's group conducted a study where the TiO_2_ was covered with a precious metal layer (Pt or Au) before the deposition of iridium nanoparticles (Figure 11a).^[182]^ They used a photoreduction method to produce conductive layer‐coated supports. Thereby, a lower resistance than the commercial 75 wt% IrO_2_−TiO_2_ catalyst could be achieved with only 39–43 wt% of precious metals. Namely, a conductivity of 44 S/cm was measured for the annealed Ir−Pt−TiO_2_, higher than the 32 S/cm for the commercial catalyst with 75 wt% IrO_2_ (Figure 11b). Bulk Pt did not oxidize and thus the catalyst maintained its conductivity under OER conditions. Furthermore, a 39 % mass activity improvement was noticed compared to the commercial catalyst (Figure 11c) although lower than previously presented ATO‐supported Ir catalysts or state‐of‐the‐art unsupported catalyst.^[138]^ Therefore, one can wonder whether the applicability of decreasing the usage of expensive Ir with layers of precious metals such as Pt and Au, already used in various sectors, is worthwhile if it does not present outstanding activity or stability improvements.

On the other hand, our group chose another approach to boost the conductivity of TiO_2_‐based support, namely the partial conversion of TiO_2_ to TiN and the formation of TiO_x_N_y_ material.[^183^, ^184^, ^185^, ^186^, ^187^] The idea is that TiN is conductive^[188]^ but slowly oxidizes under OER conditions while TiO_2_ is stable but electrically resistant and thus TiO_x_N_y_ could profit from the merits of both worlds, being conductive and stable.

A first study was conducted to ensure that TiO_x_N_y_ possesses the required characteristics of electrocatalyst support, i. e. sufficient electrical conductivity, high surface area, stability under reaction conditions and the possibility to accommodate nicely dispersed active metal.^[183]^ The conductivity of TiO_x_N_y_ before the deposition of iridium was in the range 3.3–7.8 S/cm, sufficient for electrocatalyst support. TEM pictures demonstrated that the deposition of small iridium nanoparticles (<4 nm) was achieved, leading to an increased specific surface area compared to unsupported catalysts. Consequently, the OER activity of Ir/TiO_x_N_y_ was higher than Ir‐black and IrO_2_ benchmarks. Furthermore, when TiO_2_ – nanoribbons were used as a precursor for TiO_x_N_y_, the obtained catalyst presented good stability after a 5 hours long chronopotentiometry degradation protocol (at 0.1 A/mg_Ir_). To understand the improved stability, the possible interaction between the support and the precious metal was investigated by Bele et al. The authors compared the electrochemical behavior of Ir/TiO_x_N_y_ and Ir/C catalysts during cycling (300 mV/s, 0.05–1.2, 200 cycles). In the case of Ir/C, the shape of the CV slowly evolved from metallic iridium to characteristic Ir‐oxide, namely loss of H_upd_ and apparition of oxidation peaks from 0.9 to 1.1 V_RHE_ (Figure 11d). On the other hand, the H_upd_ feature was still visible after 200 cycles for Ir/TiO_x_N_y_, suggesting the presence of metallic Ir and its resistance to oxidation, ascribed to the interaction with the support.

The next studies were focused on the impact of the support morphology as well as the strong metal‐support interaction.[^185^, ^187^] The SMSI was investigated by synthesizing the catalyst on the SEM grid and following every step of the synthesis and electrochemical characterization with IL‐SEM, XRD and XPS.^[187]^ The catalyst presented outstanding electrochemical performance, with more than three times higher activity than the commercial Ir‐black and high stability. Furthermore, the catalyst was also morphologically very stable with no visible change after severe ADT. The authors showed by XPS that the surface of the support was partially oxidized back to TiO_2_, which could explain its high stability under highly oxidizing conditions. These conclusions were supported by DFT calculations, which highlighted the strong interactions between Ir and N ions present in the support, increasing the diffusion barrier and consequently decreasing the agglomeration of Ir nanoparticles. On the other hand, the impact of the morphology was investigated by producing TiO_2_ by anodic oxidation process and varying the anodization time.^[185]^ Thereby, similar sizes of Ir nanoparticles could be deposited on TiO_x_N_y_ supports with different morphology; nanotubes (Ir−TiON_x_‐1 h), nanoparticles (Ir−TiON_x_‐6 h), and a mix of both (Ir−TiON_x_‐3 h) (Figure 11e). Morphology of the support can play an important role in the removal of the formed O_2_ bubbles and greatly enhanced the activity of catalyst.[^189^, ^190^, ^191^] After an extensive study to assure the identical chemical composition of the supports with XPS, XRD, Raman, EDX and EELS, the authors attributed the higher activity of the Ir−TiON_x_‐3 h to the specific support morphology. Indeed, the nanotubular morphology is particularly conductive compared to cluster support due to the lower particle‐to‐particle contact resistance, which is positive at low current density (around the onset potential) while the cluster‐kind support is supposedly better for bubble management and thus beneficial at high current densities.[^77^, ^189^, ^190^, ^191^] Ir−TiON_x_‐3 h presented both characteristics which resulted in low onset potential and high activity at high current densities. Furthermore, Ir−TiON_x_‐3 h also possessed the best stability (Figure 11f), ascribed to higher crystallinity of the TiO_2_ part of the support as seen in Raman spectroscopy.

Finally, a graphene‐based template (reduced graphene oxide nanoribbons, rGONRs) for the deposition of TiO_x_N_y_ was used in a follow‐up study.^[186]^ The graphene‐core covered with TiO_x_N_y_ layer could prevent the loss of conductivity due to oxidation as well as increase the surface area of the support. Therefore, three samples with a varying TiO_x_N_y_/rGONRs ratio (Low, Middle and High) were synthesized. Varying ratios led to different coverage of the graphene (Figure 11h). It was found that the positive effect of the support on the activity and stability was highly dependent on the Ti/C ratio and on the position of the Ir nanoparticles on the support. All three samples presented up to 30‐times better electrochemical performances than benchmark catalysts but also higher activity than any reported Ir‐based catalysts in the literature at the time. The best activity was recorded for the sample with the middle Ti/C ratio. The Ir nanoparticles were observed to sit at the heterojunction between TiO_x_N_y_ flakes and the rGONRs, creating a unique chemical environment. However, the most stable catalyst was the material with the most TiO_x_N_y_ presumably due to the higher presence of stable material (Figure 11g). Thereby, the fine‐tuning of morphology and composition of carbon‐ceramic support could further enhance the OER electrochemical performances through SMSI.

#### 2.2.3 Other metal oxides

Besides tin‐ and titanium‐based supports, few other transition metals have been studied as possible alternatives.[^44^, ^192^, ^193^, ^194^, ^195^, ^196^, ^197^, ^198^, ^199^, ^200^] Notably, Ta_2_O_5_ has been mixed with IrO_2_ to serve as anode in commercial electrolyzer systems with good electrocatalytic activity and stability but high content of iridium (over 70 %).[^194^, ^195^]

**Figure 12:**
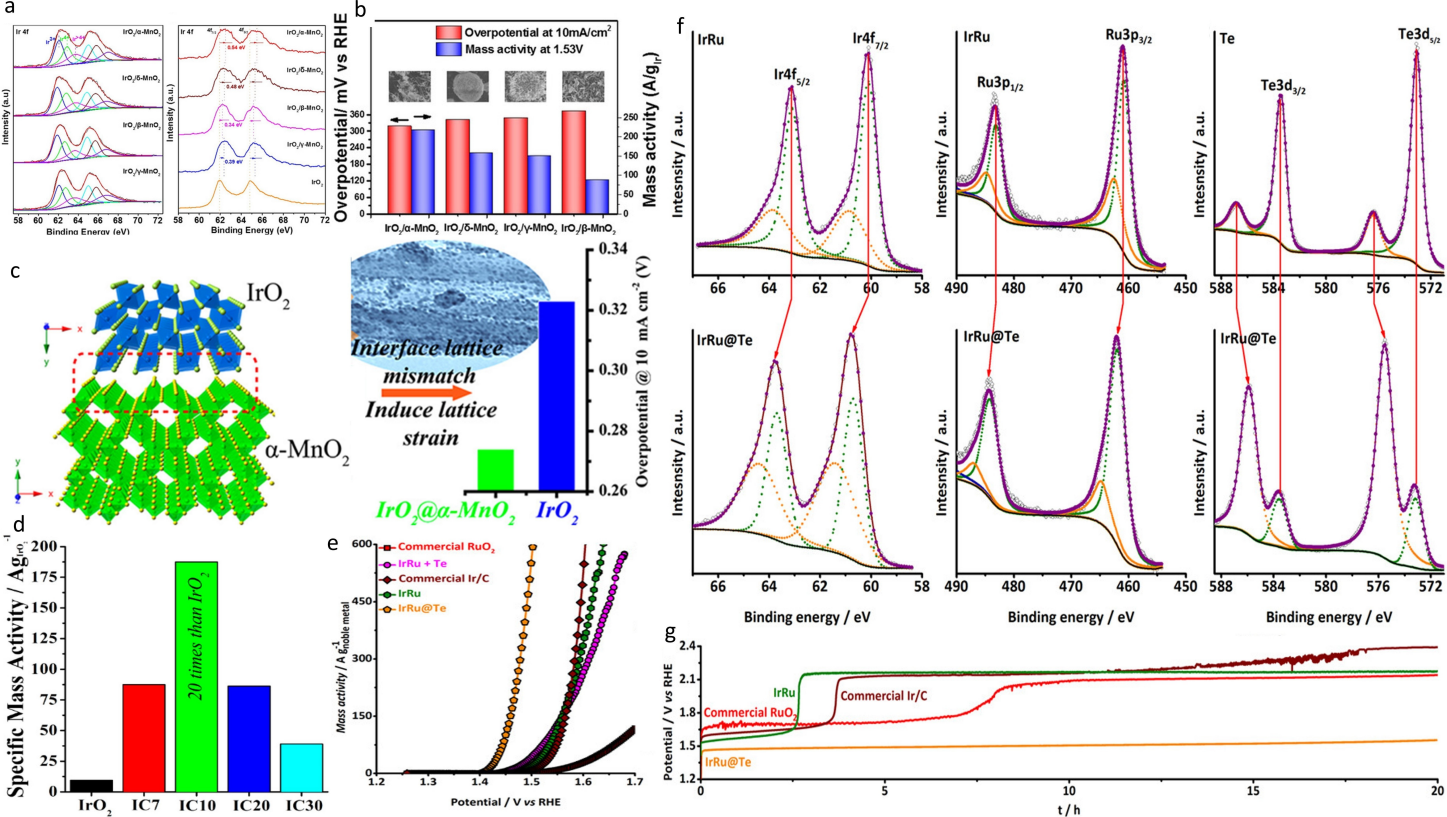
a) Fitted XPS spectra of Ir4 f (left) and Ir4 f XPS shift (right) for Ir‐based catalysts on Mn‐based support.^[200]^ b) Overpotential needed to reach 10 mA/cm^2^ (red) and mass activity at 1.53V_RHE_ for Ir‐based catalysts on Mn‐based support. Reproduced with permission.^[200]^ Copyright 2021, Wiley‐VCH. c) Schematic of the induced lattice strain due to the interaction of IrO_2_ and α‐MnO_2_ substrate and the increased activity. Modified from,^[198]^ with permission. Copyright 2017, American Chemical Society. d) Specific mass activity of Ir‐decorated Co_3_O_4_ at 1.54 V_RHE_. Reproduced with permission.^[197]^ Copyright 2018, American Chemical Society. e) Mass‐normalized polarization curves of Ir/Ru‐based catalysts.^[193]^ f) High‐resolution XPS spectra of, from left to right, Ir, Ru and Te components in IrRu@Te catalyst and unsupported IrRu catalysts.^[193]^ g) Chronopotentiometry accelerated degradation test of Ir‐based catalysts, 10 mA/cm^2^. Reproduced with permission.^[193]^ Copyright 2020, American Chemical Society.

Recently, some metals that were not expected to be stable (Co[^196^, ^197^]) or sufficiently conductive for low Ir loadings (MnO_2_,[^198^, ^199^, ^200^] 5.10^−3^ S/cm^[131]^) at OER conditions have been investigated as some specific morphology or chemical state could enhance their characteristics. Wang's group have synthesized different phases (α‐, β‐, γ‐, and δ‐) of MnO_2_ with only 5 wt% Ir.^[200]^ According to TEM and XPS measurements, the α‐phase support induced smaller Ir nanoparticles and more high‐valence state iridium (Figure 12a). Moreover, the α‐MnO_2_ showed the lowest resistance among the different supports. Consequently, Ir on α‐MnO_2_ presented a higher mass activity (Figure 12b). In previous work, Yang's group explained the improved activity of Ir/α‐MnO_2_ over unsupported and other supported catalysts from literature by the lattice strain induced in IrO_2_ crystal structure due to the lattice mismatch with the support (Figure 12c).^[198]^ This hypothesis was proven by the good agreement between the lattice strain changes, caused by different IrO_2_/MnO_2_ ratios, and the corresponding change in OER activity. If the lattice strain increases (less IrO_2_ or smaller nanoparticles), the turnover frequency of the catalysts increases too. Furthermore, with smaller nanoparticles, a charge transfer between IrO_2_ and the support can result in better activity, alike a lower Ir oxidation state in IrO_x_ on ATO.^[138]^

A similar study was conducted with one‐dimensional Co_3_O_4_ nanorods as a support.^[197]^ Co materials are not stable under the corrosive OER environment in acidic media and thus the authors varied the atomic loading of Ir during synthesis to study the minimum amount required to stabilize the Co_3_O_4_ nanorods. An Ir loading of 5 at% (named IC5) did not protect the substrate during ADT while 30 at% induced the loss of the support nanorod‐morphology and a gradual decrease of activity is visible during ADT. On the other hand, 7–20 at% of Ir on Co_3_O_4_ could sustain 50 000s at 10 mA/cm^2^. Nonetheless, the recorded activity of all samples was several times higher than the mass activity of IrO_2_ benchmark (Figure 12d). The activity improvement was attributed to the synergistic effect of two different metallic cations as revealed by XPS measurement. Finally, the importance of a one‐dimensional substrate was demonstrated by comparing it with Ir/Co_3_O_4_ mixture with the same atomic ratio. The latter could not sustain the ADT, highlighting the role of support morphology and rational design of precious metal supported catalysts.

The interaction between oxide support and the precious metal nanoparticles has also been revealed with IrRu alloy on Te.^[193]^ Indeed, XPS measurements and DFT calculation disclosed the strong electronic coupling between the support and the alloy, with an upshift of the density of state towards the d‐band centre (Figure 12f). It, once again, led to the improvement in activity and stability compared to the benchmark (Figure 12e and 12 g) and the mixture of unsupported IrRu and Te due to the suppression of the overoxidation of Ir and Ru.

Theoretically, materials based on transition metal oxides seem promising supports for OER electrocatalysts thanks to their intrinsic stability under oxidative conditions. Moreover, the low intrinsic conductivity can be tuned with fine doping, sufficient iridium loadings or appropriate morphology. In addition, they boost the iridium activity and stability through SMSI. In reality, the stability of these supports, notably doped ones, is not well understood and several studies present opposing results. Furthermore, the intrinsic conductivity does not seem to be as important in the RDE setup where thin films are employed but could become an issue for the thicker film in PEMWE. Also, the limited surface area of these materials would lead to even thicker films than what is currently used and is definitely one possible parameter for improvement.[^136^, ^146^, ^201^]

### 2.3 Other supports

Metal oxides are arguably the most studied class of materials as potential supports for OER in acidic media as they usually present good stability under corrosive environments. Nevertheless, some other materials, i. e. carbides and nitrides, are promising and will be discussed in the following sections.

#### 2.3.1 Metal Nitrides

**Figure 13:**
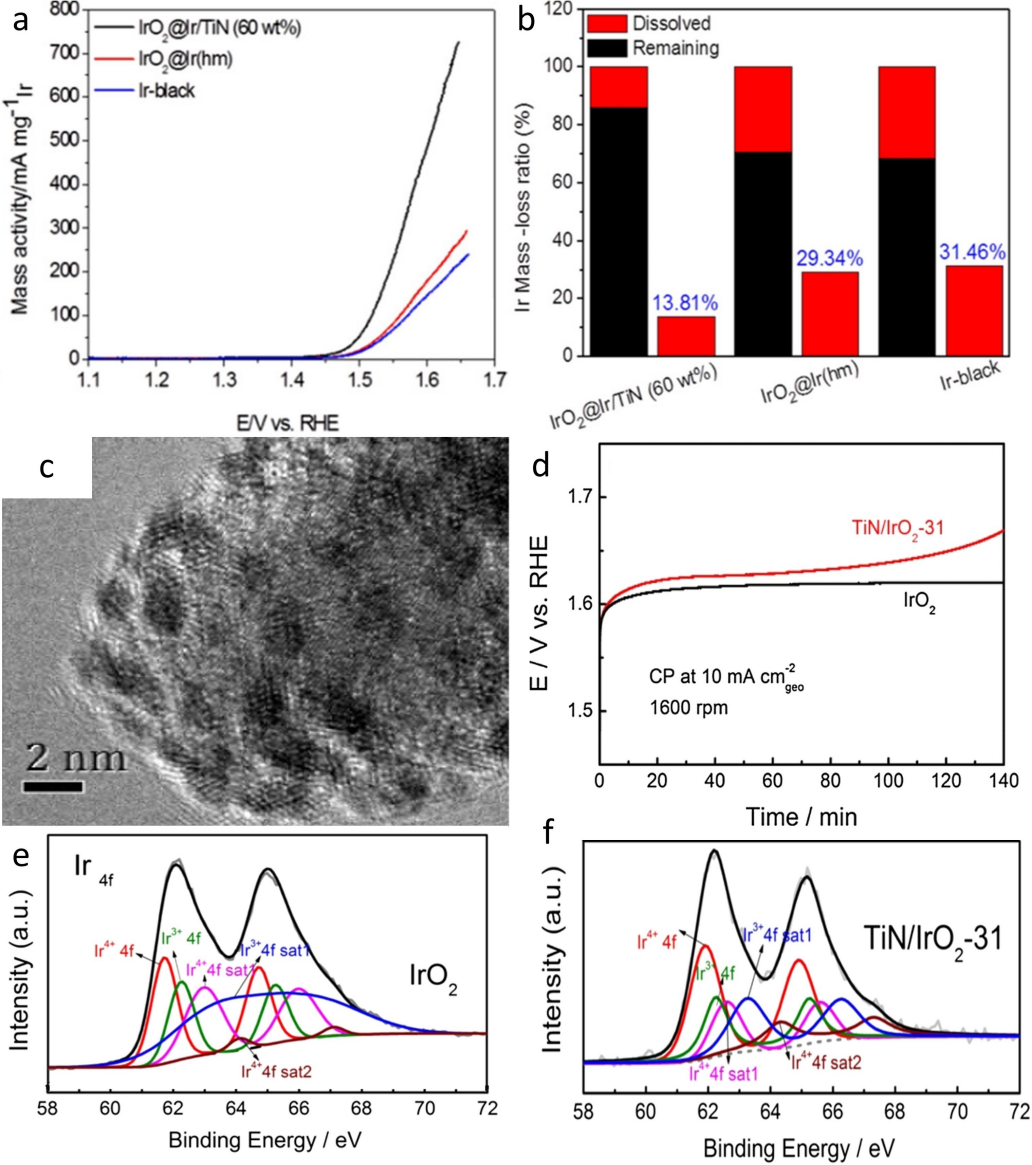
a) Mass‐normalized polarization curves of Ir‐based catalysts.^[208]^ b) Ir mass‐loss ratio based on the initial Ir mass. Reproduced with permission.^[208]^ Copyright 2018, American Chemical Society. c) HR‐TEM picture of IrO_2_/TiN (31 wt%).^[209]^ d) Chronopotentiometry accelerated degradation test of Ir‐based catalysts, 10 mA/cm_geo_^2^.^[209]^ e) HR‐XPS spectra of Ir4 f of IrO_2_.^[209]^ f) HR‐XPS spectra of Ir4 f of IrO_2_/TiN (31 wt%). Reproduced with permission.^[209]^ Copyright 2020, Springer Nature.

Metal nitrides usually possess enhanced electrical and electrocatalytic properties compared to their metal oxide counterparts.^[202]^ They can also easily form core‐shell structures during synthesis.[^203^, ^204^] One previously mentioned metal nitride as potential support in acidic media is based on titanium, i. e. TiN. It possesses high conductivity and sufficient resistance to oxidation;[^205^, ^206^, ^207^] therefore Li et al. investigated the electrochemical performances of IrO_2_@Ir nanoparticles on TiN support (IrO_2_@Ir/TiN (60 wt%)).^[208]^ The authors recorded a mass activity of 480.4 mA/mg_Ir_ at 1.6 V_RHE_ for their supported catalyst, being 2.74 and 3.32 times the activity of the IrO_2_@Ir (homemade) and Ir black unsupported counterpart, respectively (Figure 13a). The better activity was ascribed to the negative shift of the electronic binding energy of Ir species in the Ir4 f XPS spectra. This shift is caused by the electron transfer from TiN to Ir. In terms of stability, IrO_2_@Ir/TiN lost 38.1 % of activity after 6 hours at 10 mA/cm^2^ compared to 69.7 % and 73.0 % for IrO_2_@Ir (homemade) and Ir black, respectively. The better durability of the catalyst was studied based on two common degradation processes, the oxidative dissolution, and the agglomeration of Ir species. The dissolution was studied by ICP‐OES and a clearly lower Ir dissolution was visible for the TiN‐containing sample, attributed to the electron transfer from TiN to Ir which inhibits the formation of (IrO_4_)^−^ ions (Figure 13b). The aggregation of particles was analyzed with TEM images and, even if aggregation happened in all samples, the lowest increase was recorded for the supported catalyst. Thus, according to the authors, the TiN support effectively increased the activity thanks to the electron‐transfer from the support to the precious metal. This transfer also helped to boost the stability by inhibiting the oxidative dissolution in addition to the anti‐aggregation impact due to anchored nanoparticles on the support.

Years later, Zhang et al. recorded an improved mass activity of 874 A/g_Ir_ at 1.6 V_RHE_ for IrO_2_/TiN (31 wt% Ir). TiN support possesses a mesoporous, high‐surface‐area structure allowing a nice dispersion of small IrO_2_ nanoparticles (1.41±0.19 nm).^[209]^ The good atoms efficiency of such small particles partially explained the 5‐times higher activity than that of IrO_2_ benchmark. In addition, the authors compared the scattering of IrO_2_ nanoparticles on the support with strawberry‐like seeds (Figure 13c). Consequently, a higher number of unsaturated iridium atoms were exposed and available as active sites, which helped to improve the activity. However, the stability of the catalyst was found to be worse than IrO_2_ by chronopotentiometry and chronoamperometry measurements, oppositely to Li's work (Figure 13d).^[208]^ The difference could be explained by the different IrO_2_ chemical states. Indeed, in the previously discussed work,^[208]^ a negative shift of the electronic binding energy was observed for iridium nanoparticles on TiN, meaning that iridium was in a lower oxidation state when supported on TiN. In Zhang's work, the opposite was reported with a higher ratio of Ir4+/Ir3+ found in IrO_2_/TiN than in IrO_2_ (1.9 : 1 vs 1.4 : 1)^[209]^ (Figure 13e and 13 f). A difference in particles size and morphology could also explain the observed stability difference. Nonetheless, the chemical effect of TiN support seems to be different in both cases and further study should be conducted to reveal the impact of TiN on the oxidation state of IrO_2_ nanoparticles with the different particle size.

Another nitride support was based on silica, Si_3_N_4_, but only a slight enhancement of electrochemical performances was recorded.^[210]^ It was most likely due to the low interaction between the support and IrO_2_ as well as the low conductivity of Si_3_N_4_ alone. Other nitride supports for Ir‐based catalysts were investigated in alkaline media and are thus out of the scope of this review.[^211^, ^212^]

#### 2.3.2 Metal Carbides

**Figure 14:**
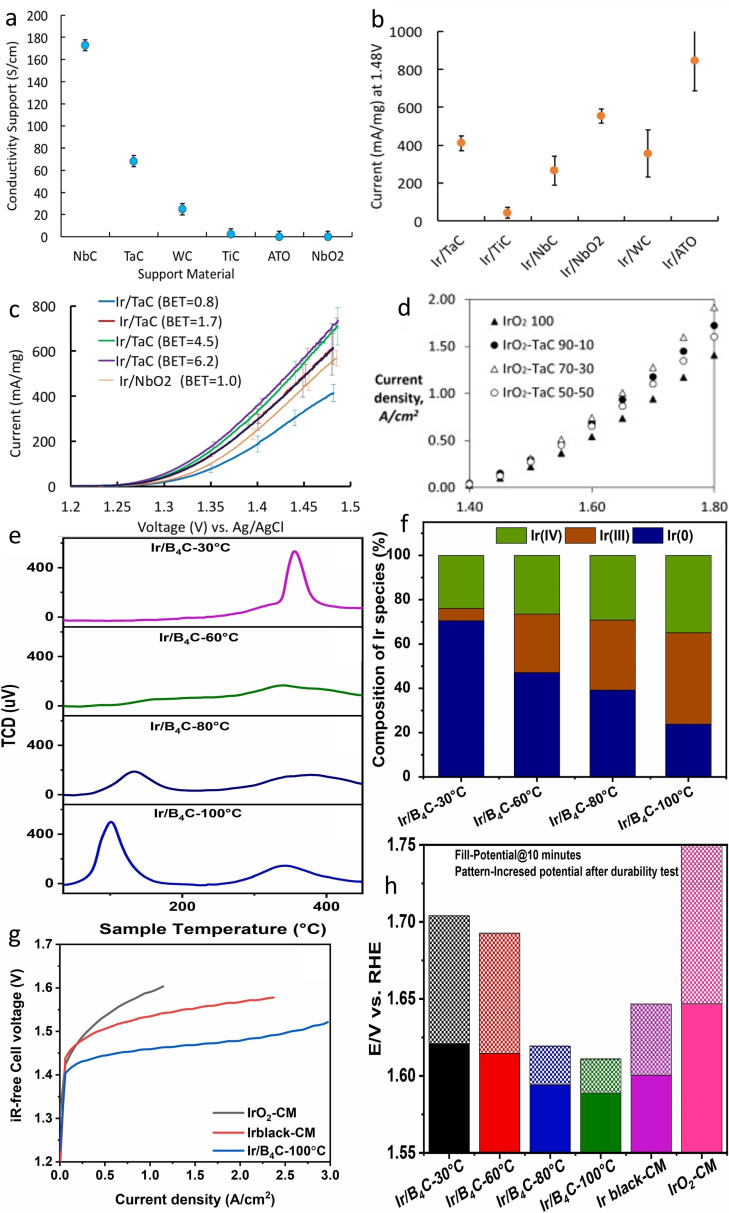
a) Conductivity of the different studied supports.^[218]^ b) Mass‐activity of Ir‐based catalysts at 1.48 V_Ag/AgCl_.^[218]^ c) Mass‐normalized polarization curves obtained for Ir/TaC and Ir/NbO_2_ catalysts with various support surface areas. Reproduced with permission.^[218]^ Copyright 2017, Elsevier. d) Load curves of PEMWE with different anode catalysts at 130 °C. Reproduced with permission.^[220]^ Copyright 2014, Elsevier. e) H_2_‐temperature programmed reduction profiles of Ir/B_4_C catalysts synthesized at various reduction temperatures.^[224]^ f) Iridium species composition obtained by XPS spectra of Ir4 f in Ir/B_4_C synthesized at various reduction temperatures.^[224]^ g) iR‐free polarization curves of a PEMWE single cell of Ir‐based catalysts.^[224]^ h) Potential reached by Ir‐based catalysts after 10 min (fill) and 3 h (pattern) of a chronopotentiometry ADT at 10 mA/cm^2^. Reproduced with permission.^[224]^ Open access 2021.

Many transition metal carbides fulfill the requirements of electrochemical support, i. e. high chemical and mechanical stability, good electrical conductivity, and sufficient surface area.[^213^, ^214^] One of the first carbide materials investigated as possible support for OER in acidic media was, again, based on titanium.[^215^, ^216^, ^217^, ^218^] The intrinsic stability of this metal makes it an interesting first choice to study. Thereby, Zhai et al. prepared Ir/TiC and inspected the suitability of TiC as an OER support.^[215]^ The chemical and electrochemical stability of the substrate under OER conditions as well as the improved activity compared to Ir‐black makes TiC a favorable candidate to support Ir in PEMWE. Nonetheless, to our knowledge, only two studies, besides the two from Zhai's group,[^215^, ^216^] were conducted with TiC supported iridium under acidic OER conditions.[^217^, ^218^] Fuentes et al. used TiC as support for Pt−Ir nanoparticles to successfully synthetize a bifunctional catalyst for regenerative fuel cell with higher round‐trip efficiency than previously presented URFC bifunctional catalysts.^[217]^ On the other hand, Karimi and Peppley studied TiC as a possible OER catalyst support along with other carbide materials.^[218]^ They based their work on previously proposed carbide‐based substrates such as TiC, TaC, NbC, WC and other materials such as NbO_2_ and ATO. First, the potential supports were compared based on conductivity study and surface area measurements. The BET measurements were similar for WC, TaC, NbO_2_ and NbC with values between 0.8 and 1.7 m^2^/g while ATO and TiC presented BET of 34.4 and 28.3 m^2^/g, respectively. The highest BET of the two latest could be due to their significantly lower particles size (nm vs μm for the four other materials). The conductivity measurement showed a different trend with the conductivity of the two oxide‐based supports (ATO and NbO_2_) and TiC being 10 S/cm while the three other carbides possessed a conductivity around 25, 68 and 173 S/cm for WC, TaC and NbC, respectively (Figure 14a). However, after deposition of 20 wt% Ir, all the samples presented similar conductivity, ranging from 0.3 S/cm (NbO_2_) to 10 S/cm (TaC). The decrease in conductivity of the carbides (NbC, TaC, WC) might be due to their oxidation during the ethylene glycol‐based synthesis. Therefore, the authors emphasized that, in their opinion, a good surface area is a more important parameter than the intrinsic conductivity of the support. Afterwards, the activity of the Ir‐supported catalysts was compared and Ir/ATO, Ir/NbO_2_ and Ir/TaC were found to be the best performing catalysts (Figure 14b). The poor performances of other carbides were not assessed by the authors except for Ir/TiC. The authors explained the lowest activity of Ir/TiC by the lowest OER activity of TiC alone compared to other support materials without Ir. In addition, the authors claimed that TiC could negatively affect the Ir activity, oppositely to Zhai's work.[^215^, ^216^] Henceforth, the materials’ intrinsic OER activity could be an important parameter for the selection of the support, according to the two authors. Finally, the importance of the support surface area was studied by ball‐milling TaC and NbO_2_ to increase their surface area before iridium deposition. A 50 % improvement in OER activity was recorded for Ir/TaC with a 6‐times higher BET of TaC (Figure 14c). Therefore, the authors proposed the following important order of support properties: Support OER Activity ∼ Support Surface Area≫Electrical Conductivity. It should be kept in mind that no comparison to benchmark was performed during the study and the OER performances were presented against Ag/AgCl, which complicated the comparison to literature. Nonetheless, among the studied carbides, TaC was the most promising. Ir/TaC was also shown to be a promising electrocatalyst in a PEMWE by Polansky et al.[^219^, ^220^] After a first study assuring the suitability of TaC as an electrocatalyst support,^[219]^ Polansky et al. synthesized IrO_2_ on TaC with 50, 70 and 90 wt%.^[220]^ Such a high loading was necessary to ensure good conductivity as well as prevent the passivation of TaC into NaTaO_3_.^[219]^ The performances of the three samples and IrO_2_ benchmark were then analyzed in a PEMWE between 1.4 and 1.8 V at various temperatures (90°, 110°, 120° and 130 °C).^[220]^ The supported catalyst outperformed the IrO_2_ benchmark. Among the supported catalysts, IrO_2_ 70 wt%/TaC was found to present the highest current densities (36 % improvement compared to IrO_2_ at 1.7 V and 130 °C, Figure 14d)), along with the lowest charge transfer and cell resistances. In addition, no degradation was observed during the experiments, making TaC possible support to be used in real devices.

In the case of high loading of iridium, the conductivity of the support is not so primordial as it will be provided by the active compound. However, to manage a significant reduction of the iridium loading, the intrinsic conductivity of the support becomes more important and not all metal carbides present a sufficiently low resistance. For example, SiC, promising support for high‐temperature PEM steam fuel cell[^221^, ^222^] due to its high stability against phosphoric acid, possesses quite poor conductivity. Thus, Nikiforov et al. doped it with silicon, synthesizing SiC−Si (>22 % free Si) as support for iridium.^[223]^ Various iridium loadings were studied, from 0 to 100 wt% by a 10 % increment. Unfortunately, the conductivity of the doped support was found insufficient without proper IrO_2_ loading, with a conductivity 1.8⋅10^−5^ S/cm for pure SiC−Si. Nonetheless, a slight reduction of Ir loading with improved performances was still observed but 60 wt% Ir was necessary to reach sufficient conductivity.

Finally, a recent study on boron carbide was conducted by Islam et al.^[224]^ They synthesized Ir on B_4_C support and varied the reduction temperature during the synthesis. Namely, four samples were prepared at 30°, 60°, 80° and 100 °C (named Ir/B_4_C−T°) and compared to Ir‐black and IrO_2_ benchmarks. The different temperatures resulted in different particle size distribution and crystallization. Indeed, TEM pictures showed that higher temperature led to smaller Ir nanoparticles with an average diameter of 22 nm, 3.2 nm, 1.98 nm, and 1.3 nm for Ir/B_4_C_–_30, Ir/B_4_C_–_60, Ir/B_4_C_–_80, Ir/B_4_C_–_100, respectively. Similarly, H_2_‐temperature programmed reduction measurements showed that amorphous IrO_2_ was present in Ir/B_4_C_–_80 and −100 but not in the two other samples (Figure 14e). This was confirmed by XPS with an increasing Ir(III)/Ir(IV) ratio with increasing synthesis temperature (Figure 14f). Consequently, Ir/B_4_C_–_80 and Ir/B_4_C_–_100 outperformed the other homemade catalysts and benchmarks during activity tests. In addition, Ir/B_4_C_–_80 and particularly Ir/B_4_ −100 were also observed to be the most stable catalysts after a 3 h chronopotentiometry ADT (Figure 14h). However, smaller nanoparticles and more amorphous iridium should exhibit faster dissolution and thus be less stable according to theory.^[225]^ Thanks to XPS, the better durability could be explained by a strong metal‐support interaction. Indeed, a shift in the Ir4 f, as well as B1s spectra, was noticed and attributed to a charge transfer between the two materials. This shift was present in all supported samples compared to the benchmark and increased with increasing synthesis temperature, pointing out a stronger interaction at higher temperatures. This interaction was also most likely involved in better activity performances. Therefore, the support helped to stabilize the less stable but more active Ir(III), when the synthesis temperature was increased. In addition, it also prevented nanoparticles agglomeration in Ir/B_4_C_–_100 as seen in TEM picture after ADT. Last but not least, the best catalyst, Ir/B_4_C_–_100, was tested in MEA and found to outperform benchmark catalysts (Figure 14g). Thus, the authors not only presented theoretically possible support but showed its suitability in a real device.

## 3 Conclusion and Outlook

The urgent need of a worldwide shift to green energy is pressing the commercialization of PEMWE, and thus the heavy reduction of iridium loading, without which green hydrogen economy will not be reachable. One possibility is to use a material that fulfils the requirements of electrocatalyst supports, i. e. high‐surface‐area, stable under acidic and oxidative environment, and electrical conductivity to decrease iridium loading and enhance its utilization. Hereby, we have reviewed the numerous investigated materials, from the extensively studied ATO to the new and promising B_4_C, passing by the surprisingly stable graphene‐based carbon. Right now, no material is currently ready for real devices and some opposite results were even recorded, e. g. for the stability of doped‐SnO_2_. This discrepancy between the results from different laboratories underlines one of the major problems of the OER field, i. e. the lack of benchmark protocols for testing as well as for analyzing performances of the catalysts. Indeed, the attentive reader would have noticed that the OER activities were measured at 1.48, 1.51, 1.55, etc V_RHE_ or at 1 or 10 mA/cm^2^. Similarly, stability was tested at different current densities in chronopotentiometry, or by chronoamperometry or cycling in various potential windows. The absence of a benchmark protocol makes it almost impossible to reliably compare results from different laboratories and thus select the best promising supported catalysts. Thus, it is primordial for the OER community to align on specific preparation and testing protocols to be able to compare results between different groups. We encourage the community to also start using GDE‐type electrochemical cells which simulate conditions that are much closer to the real device, like high current densities, temperatures, gas flow rate, and catalyst loading.^[226]^ Furthermore, it was recently shown that using RDE set‐up can lead to erroneous conclusions about the stability of Ir‐catalysts compared to the observed stability in MEA.[^227^, ^228^, ^229^] The high mass transport in liquid electrolytes permitted by the GDE set‐up can help to break the gap between RDE and MEA results.[^226^, ^230^] Recently, Yasutake et al. have developed catalysts‐integrated gas diffusion electrodes and obtained good performances at high current densities (5 A/cm^2^) and low loading of iridium. This set‐up has the advantage of being comparable to a commercial system.^[231]^

Another exciting perspective is to study the electrocatalysts with localized electrochemical techniques like scanning electrochemical microscopy^[232]^ or in‐situ TEM^[233]^ to provide further insights to correlate chemical and structural information with activity and mechanisms. However, current state‐of‐the‐art development of these techniques still does not enable one to investigate the behaviour of powdered samples, which correspond to supported Ir catalysts for OER. Nevertheless, insights into the structural changes of the catalyst, triggered by the electrochemical perturbation, can already be obtained with atomic resolution via the identical‐location TEM approach, which is now generally well‐established and has been employed for the study of supported electrocatalysts for OER[^234^, ^235^] and ORR.^[236]^

Most of the presented materials were leading to a decrease in iridium utilization, from 10 wt% to 70 wt%, with usually enhanced performances. The improved activities and stabilities were usually ascribed to the metal‐support interactions between the iridium nanoparticles and the support. This was mostly explained by a charge transfer as seen by the shift in XPS spectrum for Ir on graphene‐based supports, (doped‐)ATO, Ti‐based supports, etc. However, the question of the intrinsic conductivity of the support material is still open as it seems that this parameter is not as important in RDE set‐up that in PEMWE. Furthermore, the conductivity of the material is heavily influenced by the iridium loading. Another parameter that will play an important role in PEMWE, but is often overlooked in RDE tests, is the bubble management of the material, especially at high current densities.

## Conflict of interest

The authors declare no conflict of interest.

## Biographical Information

*Leonard Moriau obtained his master's degree (2015) in Chemistry at the Université Libre de Bruxelles (ULB), Bruxelles, Belgium. He is currently working on his PhD in the group of Prof. Nejc Hodnik in Slovenia. His study focuses on new support for Iridium nanoparticles as an OER electrocatalysts, recycling precious metals and online dissolution of PGMs*.

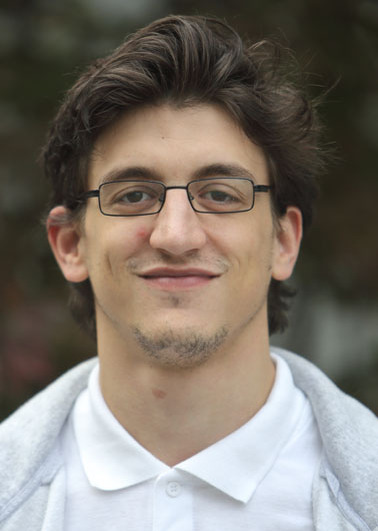

## Biographical Information

*Milutin Smiljanić obtained PhD degree in 2015 at the Faculty of Technology and Metallurgy, University of Belgrade, Serbia under the guidance of Dr. Svetlana Štrbac. He spent 18 months as a postdoctoral researcher at the Université Libre de Bruxelles, Belgium, In 2019 he joined the Laboratory of Electrocatalysis, led by Prof. Hodnik at the National Institute of Chemistry (NIC). Currently, he investigates the impact of novel support materials on the performance of electrocatalysts for fuel cells and electrolyzers*.

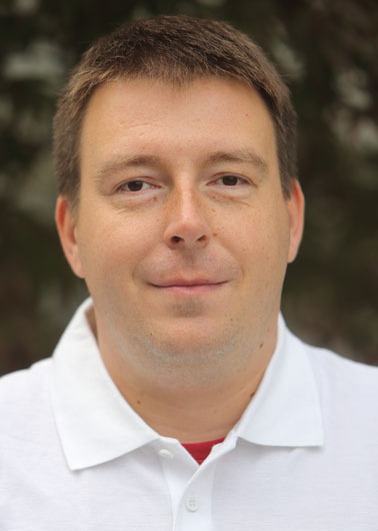

## Biographical Information

*Anja Lončar obtained her master‘s degree (2020) in Chemistry at the University of Ljubljana, Slovenia. During her master‘s study, she was a visiting student in the Electrochemical Energy Conversion group of Helmholtz Institute Erlangen‐Nürnberg for Renewable Energy (HI‐ERN), Germany lead by Dr. Serhiy Cherevko. She is currently a second‐year Ph.D. student in the Laboratory of Electrocatalysis at the National Institute of Chemistry in Ljubljana (Slovenia) lead by Prof. Nejc Hodnik. Her Ph.D. work is focusing on the stability of nanoparticulate iridium‐based electrocatalysts for oxygen evolution reaction by use of advanced electrochemical methods for degradation studies*.

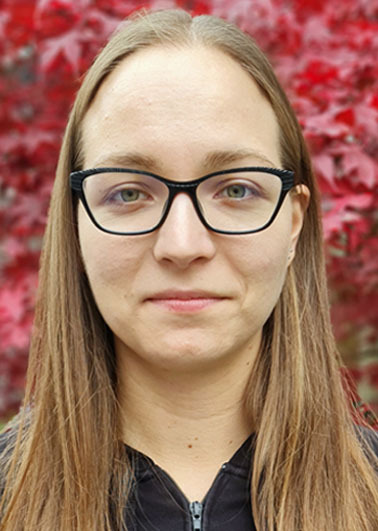

## Biographical Information

*Nejc Hodnik completed his PhD in 2013 at the National Institute of Chemistry (NIC), Slovenia. Afterwards, he was a Marie Curie Intra‐European fellow at the Max‐Planck‐Institut für Eisenforschung GmbH, Germany. In 2019, he became an associate professor at the University of Nova Gorica, obtained an ERC Starting Grant project, and started a new Laboratory for Electrocatalysis at NIC. His research focuses on nanoscale electrocatalyst degradation studies with advanced electrochemical characterization methods, synthesis, electron microscopy, recycling, etc*.